# Comparative Study of the Antimicrobial Effect of Nanocomposites and Composite Based on Poly(butylene adipate-co-terephthalate) Using Cu and Cu/Cu_2_O Nanoparticles and CuSO_4_

**DOI:** 10.1186/s11671-019-2987-x

**Published:** 2019-05-09

**Authors:** A. F. Jaramillo, S. A. Riquelme, G. Sánchez-Sanhueza, C. Medina, F. Solís-Pomar, D. Rojas, C. Montalba, M. F. Melendrez, E. Pérez-Tijerina

**Affiliations:** 1 Department of Mechanical Engineering, Universidad de La Frontera, Francisco Salazar 01145, 4780000 Temuco, Chile; 2 Department of Materials Engineering (DIMAT), Faculty of Engineering, University of Concepción, 270 Edmundo Larenas, Box 160-C, 4070409 Concepción, Chile; 3 Department of Restorative Dentistry, Endodontic Discipline, Faculty of Dentistry, University of Concepción, Concepción, Chile; 4 Department of Mechanical Engineering (DIM), Faculty of Engineering, University of Concepción, 219 Edmundo Larenas, Concepción, Chile; 5 Nanoscience and Nanotechnology Laboratory, Faculty of Physical-Mathematical Sciences, Universidad Autónoma de Nuevo León, 66451 San Nicolas de los Garza, Nuevo León México; 6 Advanced Nanocomposites Research Group (GINA), Department of Materials Engineering (DIMAT), Faculty of Engineering, University of Concepción, 270 Edmundo Larenas, Box 160-C, 4070409 Concepción, Chile; 7 Departamento de Tecnologías Industriales, Universidad de Talca, Camino a Los Niches KM 1, Curicó, Chile

**Keywords:** PBAT, Bio-nanocomposite, Copper nanoparticles, Antimicrobial activity

## Abstract

Nanocomposites and a composite based on poly(butylene adipate-co-terephthalate) (PBAT) were synthesized using commercial copper nanoparticles (Cu-NPs), copper/cuprous oxide nanoparticles (Cu|Cu_2_O-NPs), and copper sulfate (CuSO_4_), respectively. The Cu|Cu_2_O-NPs were synthesized using chemical reduction and characterized by X-ray diffraction (XRD) and transmission electron microscopy (TEM). The synthesis of Cu|Cu_2_O-NPs yielded a mixture of Cu and Cu_2_O, with metal Cu having a spherical morphology of approximately 40 nm in diameter and Cu_2_O with a diameter of 150 nm. To prepare the nanocomposites (NCs) and the composite material (MC), the NPs and the CuSO_4_ salt were incorporated into the PBAT matrix in concentrations of 1, 3, and 5% p/p via an ex situ method. Fourier transform infrared spectroscopy (FTIR), a tensile test, differential scanning calorimetry (DSC), thermogravimetric analysis (TGA), and agar diffusion assays were used for structural, thermomechanical, and antimicrobial characterization. Results showed that the reinforcements did not modify the chemical structure of the PBAT and only slightly increased the percentage of crystallization. The mechanical and thermal properties of the PBAT did not change much with the addition of fillers, except for a slight increase in tensile strength and thermal stability, respectively. The agar diffusion antimicrobial assays showed that the NCs and MCs had good inhibitory responses against the nonresistant strains *Enterococcus faecalis*, *Streptococcus mutans*, and *Staphylococcus aureus*. The MCs based on CuSO_4_ had the highest biocidal effect, even against the resistant bacteria *Acinetobacter baumannii*.

**Electronic supplementary material:**

The online version of this article (10.1186/s11671-019-2987-x) contains supplementary material, which is available to authorized users.

## Introduction

Most plastic materials are produced from fossil fuels and are practically nondegradable, which generates concerns about economic and environmental sustainability [1, 2]. Thus, the development and synthesis of biodegradable materials from another source has received much attention from the scientific community with the goal of reducing the production of petroleum-based plastics [3–5]. Biodegradable polymers have begun to play a fundamental role in solving these problems as a promising option to fossil fuels together with a new class of materials known as bionanocomposites, which, through nanotechnology, have come to possess better properties [6–10].

Bionanocomposites consist of an organic matrix in which inorganic nanomaterials are dispersed [8, 11–13]. The different morphologies and sizes of the inorganic components, such as nanoparticles, nanotubes, nanosheets, nanowires, and nanoclay, have a considerable effect on the properties of the polymer matrix. The optical, thermal, mechanical, magnetic, and optoelectronic properties are improved because of the synergy between the surface area, high surface reactivity, excellent thermal stability, and high mechanical strength of the inorganic components and the polymer matrix [14–16]. A wide range of innovations in polymer chemistry and micro- and nanofabrication techniques have driven research in polymer bionanocomposites, not only for the production of improved structures, but also for the preparation of new functional materials with interesting properties and highly sophisticated applications [17–19]. Several biopolymers of natural or synthetic origin, such as polylactic acid (PLA) [20] and poly(butylene-adipate-co-terephthalate) (PBAT), have been widely studied [21, 22].

One polymer that is currently being used as the matrix in nanocomposites is PBAT [23]. This synthetic biopolymer is a linear aliphatic biodegradable polyester based on the monomers 1,4-butanediol, adipic acid, and terephthalic acid in the polymer chain [24]. Its properties are similar to those of low-density polyethylene because of its high molecular weight and long-chain branched molecular structure, which makes it flexible [24–26]. The main limitation of PBAT is its poor mechanical strength; however, with the addition of nanosized loads, this disadvantage can be overcome thus endowing this material with multifunctional properties such as better thermomechanical properties [6, 27].

Currently, there is also an urgent need to develop bionanocomposites that can control or prevent microbial colonization by incorporating nanoparticles with known antibacterial activity into or enhancing the antibacterial properties already possessed by the polymer matrix. In the latter case, the substantial improvement in the biocidal capacity of the polymer matrix has been associated with the synergy between the two components of the bionanocomposite [28, 29]. Therefore, the polymer not only provides a support matrix for the nanoparticles, but can also improve the antibacterial performance and extend the possible applications of the bionanocomposite to meet various requirements for biomedical applications or medical devices such as endotracheal tubes and vascular and urinary catheters [30–32]. However, the use of PBAT in medical devices has not been studied extensively; only a few articles have reported the possibility of its use in some clinical applications [1].

Several investigations have reported the use of metal nanoparticles as an antimicrobial agent. The intrinsic biological property of these materials depends on several factors such as the metal involved, particle size, structure, and surface area. All possible combinations of these factors can delay antibacterial resistance [33]. Most antimicrobial studies of nanocomposites have focused on food packaging, and the biocidal activity has always targeted the same bacteria. It is not certain if the bacteria become resistant to the biocidal nanoparticles in the same way they do to drugs. Thus, one of the objectives of this work was to evaluate the antimicrobial activity of nanocomposites containing PBAT with different concentrations of Cu-NPs for potential use in the manufacture of dental implements. In addition, we performed a complete comparative study on the thermomechanical and antimicrobial properties of PBAT-based materials. PBAT nanocomposites were prepared with Cu nanoparticles at three different concentrations. Similarly, nanocomposites were prepared using Cu|Cu_2_O-NPs as load. Finally, a CuSO_4_-based composite material was prepared at the same concentrations used to prepare the nanocomposites. The biocidal activity of the nanocomposites and the PBAT composite was evaluated against *Staphylococcus aureus*, which is responsible for cutaneous infections such as folliculitis, furunculosis, and conjunctivitis; *Streptococcus mutans*, which is partly responsible for dental plaque and dental biofilm; and *Enterococcus faecalis* and *Acinetobacter baumannii*, which can cause infections that compromise humans, especially in the hospital environment.

## Materials and Methods

### Materials

PBAT (Ecoflex) used for the preparation of nanocomposites was supplied by BASF (Ludwigshafen, Germany). Its molecular structure is shown in Additional file 1: Figure S1 (supplementary material). The 99.99% pure metal Cu nanoparticles (Sigma-Aldrich, St. Louis, MO, USA) were between 100 and 200 nm in diameter. For the synthesis of the Cu|Cu_2_O-NPs, CuSO_4_ was used as a precursor, ascorbic acid (C_6_H_8_O_6_) as a reducing agent, and sodium hydroxide (NaOH) as a pH controller. In addition, CuSO_4_ (Sigma-Aldrich) was used to prepare the composite material.

### Synthesis of Nanoparticles by Chemical Reduction

A synthesis method proposed by Khan et al. [34] was used to obtain Cu|Cu_2_O-NPs. The synthesis started by dissolving CuSO_4_ × 5H_2_O in distilled water to obtain 120 mL of 0.1 M solution. Next, the 120 mL of CuSO_4_ was added to a flask immersed in a propylene glycol bath, followed by rapidly adding 50 mL of C_6_H_8_O_6_ solution. The mixture was vigorously stirred at approximately 390 rpm for 30 min while the temperature was increased to 80 °C, upon which 30 mL of NaOH solution was added dropwise and the solution was continuously agitated for 2 h. The final solution was allowed to settle overnight, and then, the supernatant liquid was removed. The concentrate was centrifuged and washed with distilled water and ethanol. Finally, the particles were dispersed using ultrasound equipment, placed in Petri dishes, and oven-dried at 60 °C overnight (see Additional file 1: Figure S2).

### Nanocomposite synthesis

To prepare the nanocomposites and the composite material, Cu-NPs, Cu|Cu_2_O-NPs, and CuSO_4_ salt were incorporated into the PBAT matrix in concentrations of 1, 3, and 5%. First, the PBAT was melted, and then, the NPs were added and mixed in a torque rheometer (model 835205, Brabender GmbH & Co. KG, Duisburg, Germany) for 7 min at 60 rpm and a work temperature of 140 °C (Additional file 1: Figure S4). The maximum load was 5% because higher loads produced fluorescence effects in the Raman spectra (Additional file 1: Figure S3).

### Characterization

The obtained nanocomposites and composite materials were characterized to study their differences with respect to the PBAT polymer. Likewise, we studied how the different concentrations of Cu-NPs, Cu|Cu_2_O-NPs, and CuSO_4_ inside the polymer affected its mechanical, thermal, morphological, structural, and bactericidal properties.

Cu-NPs and Cu|Cu_2_O-NPs were characterized via X-ray diffraction (XRD) and transmission electron microscopy (TEM). PBAT nanocomposites with Cu-NPs (NCs-PBAT/Cu) and Cu|Cu_2_O-NPs (NCs-PBAT/Cu|Cu_2_O) and the PBAT composite material with CuSO_4_ (MCs-PBAT/CuSO_4_) were characterized via thermogravimetric analysis (TGA), differential scanning calorimetry (DSC), scanning electron microscopy (SEM), Fourier transform infrared spectroscopy (FTIR), XRD, tensile tests, and antimicrobial activity assay using agar diffusion. A 100-mm × 100-mm × 1-mm plate-shaped sample of each nanocomposite was prepared so that the samples homogenized in each analysis were the same size. To obtain the plate shape, NCs-PBAT/Cu, NCs-PBAT/Cu|Cu_2_O, and MCs-PBAT/CuSO_4_ were molded using a Labtech hydraulic press (model LP-20B; Labtech Engineering Co., Ltd., Samutprakarn, Thailand) at 160 °C and 110 bars for 5 min. The preheating and cooling times were 15 min and 1 min, respectively (Additional file 1: Figure S4).

### Morphological and Structural Properties

To verify the nanometric scale of the nanoparticles and that the synthesized powders were a mixture of Cu and Cu_2_O nanoparticles, a structural analysis was performed using XRD and a morphological analysis was performed using TEM.

TEM micrographs of Cu|Cu_2_O-NPs were obtained with a JEM 1200 EX II transmission electron microscope (JEOL, Ltd., Tokyo, Japan) at a voltage of 120 kV. A sample was prepared by placing a drop of nanoparticles diluted in ethanol on a 200-mesh carbon-coated copper grid. In addition, the nanoparticles were analyzed via an electron diffraction pattern.

XRD spectra of the Cu-NPs, Cu|Cu_2_O-NPs, nanocomposites, and composite material were obtained using a Bruker Endeavor diffractometer (model D4/MAX-B; Bruker, Billerica, MA, USA). The sweep of 2θ was from 4 to 80° with a 0.02° step and counting time of 1 s. The diffractometer was operated at 20 mA and 40 kV with a copper cathode lamp (*λ* = 1.541 Å).

FTIR spectra of the nanocomposites were obtained using a Spectrum Two FTIR spectrometer (× 1720) (PerkinElmer, Waltham, MA, USA) with the attenuated total reflection (ATR) function. Each spectrum was obtained by consecutive scans in the range of 4000–500 cm^−1^ with a resolution of 1 cm^−1^.

### Mechanical Properties (Tensile Test)

Tensile tests, based on the ASTM D638 standard, were carried out on a smarTens universal testing machine (005 model; Emmeram Karg Industrietechnik, Krailling, Germany) at a test speed of 50 mm/min and a load cell of 1 kN. The V-type specimens were manufactured by compression at molding temperatures of 160 °C. The preheating, pressing, and cooling times were 7, 5, and 1 min, respectively. Five samples of each NC and MC under study were manufactured, and the tensile strength, ultimate elongation percentage, and modulus were obtained.

### Thermal Properties

TGA was carried out using a TG 209 FI Iris® thermo-microbalance (NETZSCH-Gerätebau GmbH, Selb, Germany). The samples, ranging from 3 to 10 mg, were placed in aluminum crucibles, which were then loaded into the instrument. The mass change as a function of temperature was measured by heating the samples from 20 to 600 °C at a rate of 10 °C/min under a N_2_ atmosphere.

DSC analysis was performed using a NETZSCH differential scanning calorimeter (DSC 204 F1 model). Nanocomposite samples (5–10 mg) were placed in sealed aluminum crucibles, which were heated from 25 to 200 °C at a rate of 10 °C/min under a constant N_2_ flow rate of 20 mL/min. The melting temperature (*T*_m_) was obtained from this DSC analysis.

### Antimicrobial Activity Assays of the NCs and MC Using Agar Diffusion

The antibacterial activity of the nanocomposites and composite material based on Cu-NPs, Cu|Cu_2_O-NPs, and CuSO_4_ was determined using the diffusion growth kinetics method in agar. The analysis was carried out in two stages following the protocol of Jaramillo et al. [35]. Four strains of bacteria were used: two clinical strains, *A. baumannii* (ABA 538) isolated from an intrahospital infection and *E. faecalis* (6.4) from an oral infection, and two collection strains, *S. aureus* (ATCC) and *S. mutans* (ATCC 25175).

The first stage consisted of a qualitative evaluation of antibacterial activity to select which of the three concentrations of nanocomposites and composite material to use to perform the quantitative tests to reduce the experimental design because using three load concentrations would be very expensive. After the evaluation tests, the sample with the load percentage that showed the best contact inhibition was selected. To perform the qualitative tests, *A. baumannii* (ABA 538), *E. faecalis* (6.4), *S. aureus* (ATCC), and *S. mutans* (ATCC 25175) were separately seeded on a trypticase soy agar (TSA) and incubated overnight at 37 °C. After culturing, a well-isolated colony was selected and transferred to a tube containing 4–5 mL of TSA broth using an inoculating loop. The broth was incubated again overnight at 37 °C until it reached or exceeded the turbidity of 0.5 on the McFarland scale. The turbidity of the inoculum then was adjusted with saline solution up to 0.5 on the McFarland scale using a turbidimeter. The prepared suspension contained approximately 1 × 10^8^ CFU/mL, which was diluted to 1:10 to obtain a final inoculum concentration of 10^7^ CFU/mL. TSA plates were seeded uniformly with each inoculum. Then, sheets (10 × 10 mm^2^) of the nanocomposites and composite material at concentrations of 1, 3, and 5%, plus a PBAT control, were placed on the surface of the TSA plates and checked to make sure that they adhered well. Finally, the plates were placed in an oven and incubated at 37 °C for 24 h to observe the inhibition of the PBAT samples.

The second stage of the growth kinetics method consisted of quantitative tests performed on only those nanocomposites and composite material where contact inhibition was evident in the qualitative test. To maintain sterility, the tests were carried out using a 1200 Series Type A2 Biological Safety Cabinet (ThermoFisher Scientific, Waltham, MA, USA). First, the samples were preconditioned by placing them inside sterile Petri dishes and bringing them to the biosafety cabinet where they were exposed to UV light for 15 min on each side. Next, 24-h bacterial cultures of each strain were adjusted to a turbidity of 0.5 on the McFarland scale to subsequently create six serial dilutions (1, 2, 3, 4, 5, and 6). An initial count was performed on dilutions 4, 5, and 6 (in triplicate) to determine the count at time zero.

Wet chambers, one for each evaluation time (2, 4, 6, and 8 h) and for each strain, were prepared by placing sterile gauze moistened with sterile distilled water into sterile Petri dishes. Then, a sterile slide was placed inside each wet chamber such that the upper side did not touch the wet gauze. Next, three 1 × 1-cm^2^ sheets of the nanocomposites and composite material, and PBAT sheets as controls, were placed in the chambers with the help of a sterile clamp. Dilution (20 μL) was deposited on each square sheet, and the chambers were incubated at 37 °C for 2, 4, 6, and 8 h.

After incubation, the wet chambers were extracted, and each polymer sheet was deposited inside a Falcon tube with 1 mL of sterile distilled water. The tubes were vortexed for 2–5 min [35]. Three dilutions were made from the product in the Falcon tubes. Petri dishes containing TSA were divided into four parts. Approximately three to five drops (corresponding to 20 μL) of each of the three dilutions and one drop of the undiluted Falcon tube contents were placed in the quadrants. The agar plates had to be completely dry so that the drops were absorbed almost instantaneously. The plates were then incubated at 37 °C for 24 h followed by a colony count with a colony counter. The data obtained were multiplied by the dilution factor used and plotted in graphs using the logarithm function or survival percentage.

## Results and Discussion

Rheometry is used to obtain dynamic measurements of the rheological properties of nanocomposites under conditions close to the actual conditions under which the nanocomposites were processed. For this, measurements were made to control the changes in viscosity during melt mixing. The results of these measurements are shown in Additional file 1: Figure S5. The increase in the motor torque is related to the melting viscosity of the polymer [21, 36], and the values start to be constant after 4 min of mixing. This confirms that the mixing time of 7 min established in this work was enough to achieve complete mixing.

The torque values for the PBAT and NCs-PBAT/Cu 1% matrix were around 19.86 N m. The curves (Additional file 1: Figure S5) indicate that 1% concentration of Cu-NPs had little effect on the mechanical properties of the matrix, but lower equilibrium torque values of 18.4 and 17.4 N m were obtained for NCs-PBAT/Cu 3% and NCs-PBAT/Cu 5%, respectively. These results clearly imply that the processability of NCs-PBAT/Cu was improved with respect to the PBAT matrix [37]. Similar results were obtained with the mixture of NCs-PBAT/Cu|Cu_2_O, where the equilibrium torque value decreased with the increase in load percentage to 3%, but the 5% load yielded a value very close to that of the 1% load of Cu|Cu_2_O-NPs. The equilibrium torque values were 19.39, 19.07, and 19.37 Nm for 1, 3, and 5%, respectively. For the MCs-PBAT/CuSO_4_ mixture, the equilibrium torque values increased as the load of CuSO_4_ increased, i.e., 18.71 N m for 1%, 19.16 N m for 3%, and 19.79 N m for 5% load. This behavior can be attributed to the size of the CuSO_4_ crystals. Simultaneously, Additional file 1: Figure S5 shows that the equilibrium torque of all nanocomposites and composite material was stable with increasing mixing time, indicating that thermal decomposition did not occur in the mixer, probably because the nanoparticles decrease the cohesion forces between the polymer chains and most likely perform self-lubrication in the mixing process [37].

### Morphological and Structural Properties

**Fig. 1:**
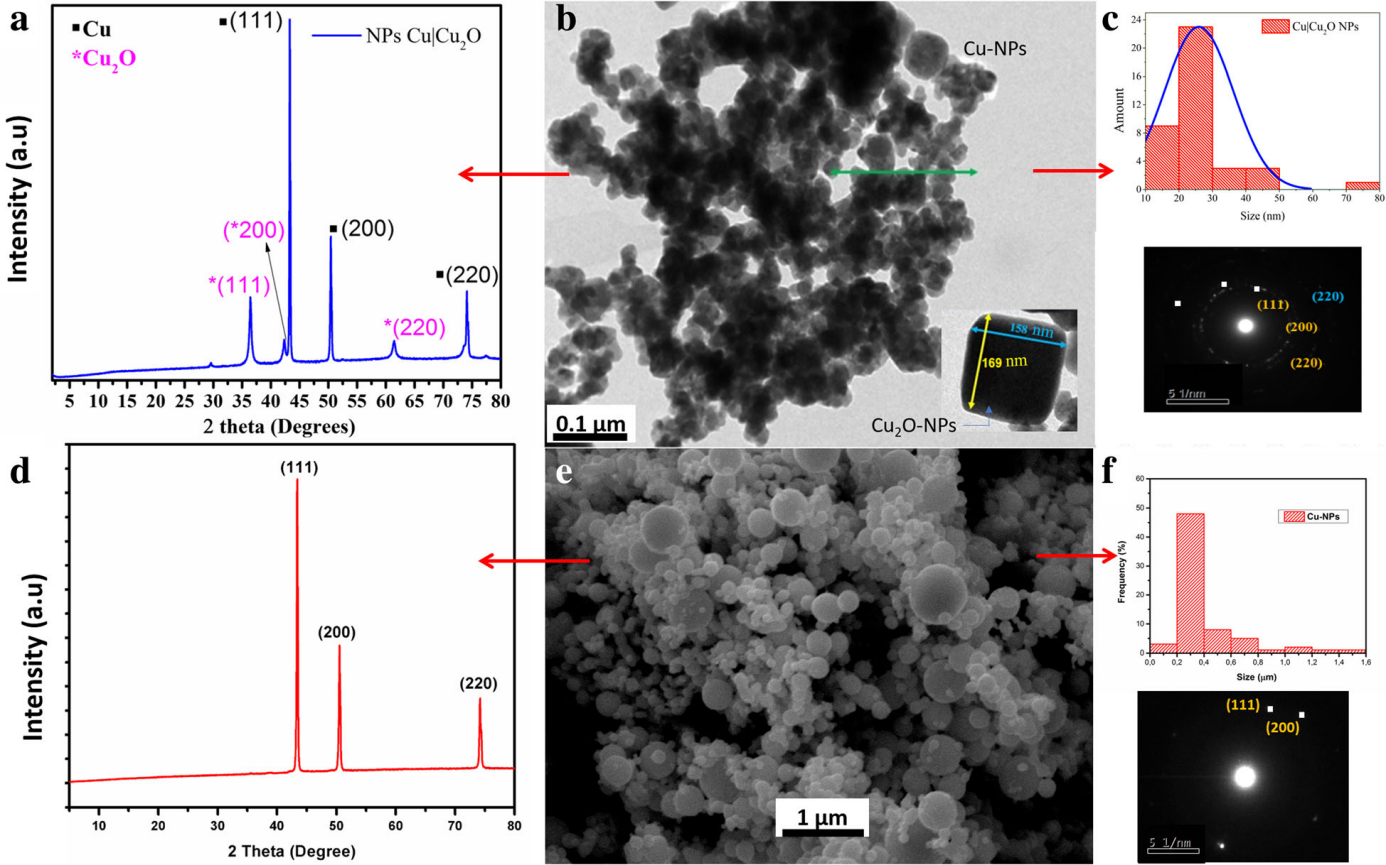
**a** XRD of Cu and CuO2 nanoparticles synthesized. **b**, **c** TEM image, size distribution, and diffraction pattern of the synthesized nanoparticles. **d** XRD of Cu nanoparticles. **e**, **f** TEM image, size distribution, and diffraction pattern of Cu nanoparticles

First, the nanoparticles obtained by chemical reduction were analyzed. The results of the synthesis of Cu|Cu_2_O-NPs are shown in Fig. 1b. The TEM micrograph shows a mixture of spherical particles and polyhedral particles. The average diameter of the spherical nanoparticles was 26 nm (Fig. 1c), while the diameter of the polyhedral nanoparticles ranged between 80 and 160 nm. The composition of these nanoparticles was determined by selected area electron diffraction (SAED) (Fig. 1c), which found phases corresponding to metal Cu and Cu_2_O. This finding was corroborated by the diffractogram shown in Fig. 1a. Six diffraction peaks were clearly observed at 2θ = 36.3°, 42.17°, 43.42°, 50.63°, 61.47°, and 74.37°. Because the nanoparticles were synthesized by chemically reducing CuSO_4_ to CuO, the diffraction peaks were verified by the data for Cu in the X’Pert HighScore database of X-ray powder diffraction patterns. We observed that the peaks at 2θ = 43.2°, 50.63°, and 74.37° belong to metal Cu diffraction planes (111), (200), and (220). The other three peaks show that the synthesized nanoparticles contained more than one substance, so the diffraction pattern is a combination of both. Wijesundera [38] analyzed thin films of Cu_2_O using XRD and showed that the planes diffracted at 2θ = 36.3°, 42.17°, and 61.47° correspond to the Miller indexes (111), (200), and (220). These indexes belong to a face-centered cubic structure (FCC) that corresponds to a part of the central area of an antifluorite structure, which agrees with the structure of Cu_2_O, in accordance with the findings of the SAED analysis.

Wang et al. [39] found that during the synthesis of Cu-NPs by chemical reduction, the size of the particles ranged between 100 and 150 nm. They used C_6_H_8_O_6_ as the reducing agent and poly(vinylpyrrolidone) (PVP) as the surfactant. The faces did not correspond to those of Cu_2_O because the PVP helped stabilize the growing seeds, thus avoiding their oxidation. However, the objective of our investigation was to synthesize Cu_2_O NPs, which can be achieved by chemical reduction without the use of a stabilizing agent such as PVP.

The Cu-NPs used in the preparation of the nanocomposite were spherical with a diameter ranging between 100 and 200 nm (Fig. 1e, f). In the XRD pattern for Cu-NPs shown in Fig. 1d, the three peaks clearly observed at 43.60°, 50.72°, and 73.95° correspond to the crystalline planes (111), (200), and (220), respectively. The cubic crystal structure with an Fm3m space group (JCPDS No.85-1326) [55] is in accordance with the structure found by SAED analysis (Fig. 1d).

The metal particles used in our study were obtained by means of a mechanical grinding system, according to the supplier. The disadvantage of this method is that a small percentage of particles (~ 10%) are larger than 500 nm. However, this did not negatively affect the objectives of our investigation. Below, we demonstrate how this dispersion affected the thermomechanical properties of the PBAT matrix. Importantly, mechanical grinding methods do not use precursors or stabilizers, as is the case with wet synthesis methods, which are known as chemical reduction methods. Therefore, the surface of Cu-NPs obtained by grinding is not passivated by the adsorption of molecules from either a stabilizer or a reaction by-product. Thus, these Cu-NPs, while not substantially improving the mechanical properties of the polymer, do not degrade them either. However, the antimicrobial properties must be improved because the migration of Cu^2+^ is facilitated on nonpassivated surfaces.

**Fig. 2:**
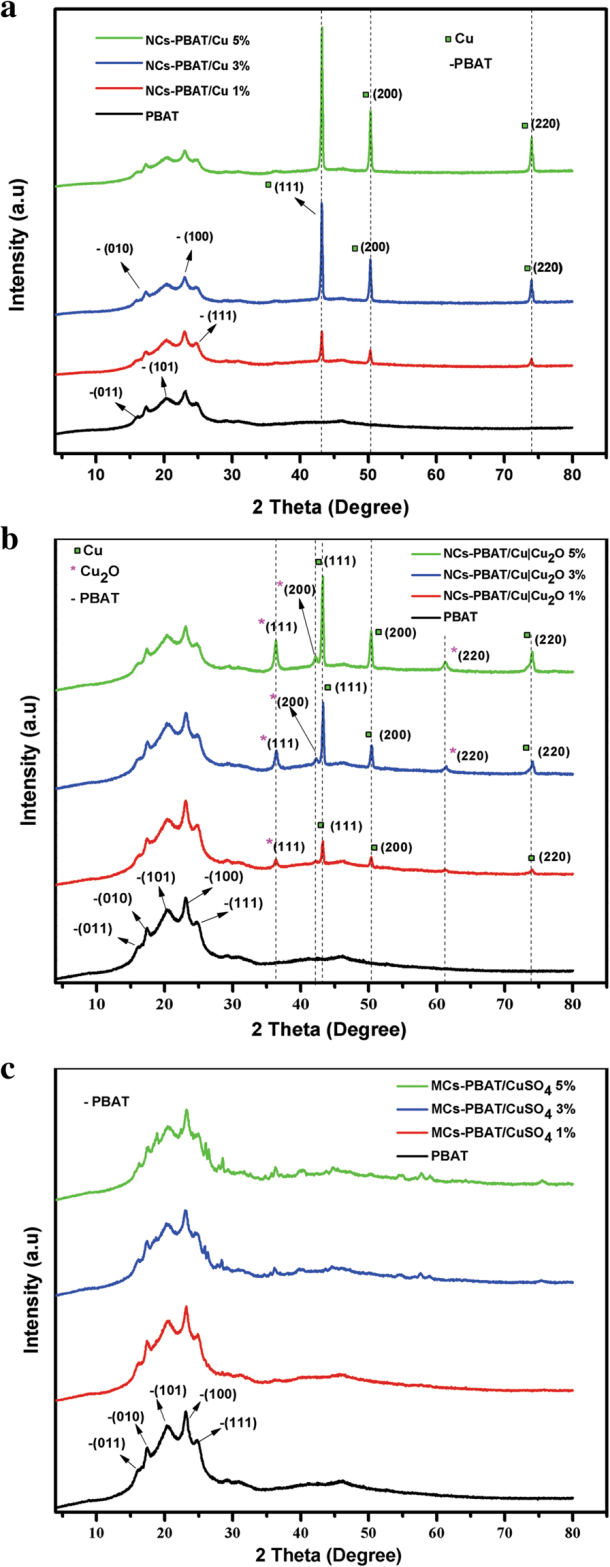
Diffractogram of PBAT, NCs-PBAT/Cu, NCs-PBAT/Cu|Cu_2_O, and MCs-PBAT/CuSO_4_

Figure 2 presents the XRD spectra of the NCs-PBAT/Cu (Fig. 2a), NCs-PBAT/Cu|Cu_2_O (Fig. 2b), and MCs-PBAT/CuSO_4_ (Fig. 2c). Figure 2c was prepared at three concentrations (1, 2, and 3% *w*/*w*). These diffractograms were compared with that of the PBAT polymer matrix to demonstrate the effect of the loads on the polymer structure. The PBAT diffractogram showed a diffraction pattern with five diffraction peaks at 2θ = 16.1°, 17.3°, 20.2°, 23.1°, and 25°, corresponding to planes (011), (010), (101), (100), and (111), respectively. This analysis revealed the existence of crystallinity in the polymer matrix. The characterization of PBAT by Arruda et al. [40] using XRD also found the same five diffraction peaks at the same angles as those found in this investigation, corresponding to the same planes.

The diffractograms of the nanocomposites with Cu-NPs loads are shown in Fig. 2a. The 2θ signals at 43°, 50°, and 74° are characteristic of the planes (111), (200), and (220) of the FCC structure of Cu with an Fm3m space group (JCPDS No.85-1326) [41]. No phases corresponding to CuO or Cu_2_O were observed in the diffractogram of NCs-PBAT/Cu, so we concluded that the nanoparticles were not oxidized during the synthesis of the nanocomposite. In addition, the diffractograms show that the nanoparticles did not affect or modify the structure of the PBAT and that the intensity of the peaks is directly proportional to the load percentage of the Cu-NPs. The diffractograms of the NCs-PBAT/Cu|Cu_2_O have six characteristic peaks at 2θ = 36.4°, 43°, 42.4°, 50°, 61.5°, and 74° (Fig. 2b). According to the literature and the analysis of the nanoparticles, only three correspond to metal Cu and the peaks at 36.4°, 42.4°, and 61.5° belong to Cu_2_O, according to the spectrum of this type of nanoparticle shown in Fig. 1a [35].

The diffraction peaks corresponding to the Cu|Cu_2_O-NPs reinforcements became more intense as the concentration increased inside the matrix, but the peaks belonging to the crystalline zone of the polymer decreased slightly in intensity with the incorporation of loads. Chivrac et al. [42] reported similar results in a study using loads of nanoclays in PBAT. They suggested that there was no significant transcrystallinity at the load-polymer interface, and therefore, there were no changes in the crystalline structure of the polymer. However, the decrease in the intensity of the diffraction peaks of the PBAT with the increase in the concentration of loads in the matrix indicates a drop in the crystallinity of the PBAT. Therefore, the loads hinder the crystalline growth of the PBAT. This could explain the slight decrease in the diffraction peaks belonging to the PBAT with the increase in Cu|Cu_2_O-NPs.

Figure 2c shows the XRD spectra of MC-PBAT/CuSO_4_ for the three concentrations of CuSO_4_ of 1, 3, and 5%. The addition of the 1% CuSO_4_ load did not generate changes in the polymer. The 3 and 5% CuSO_4_ load curves show only a minimum increase in the intensity of the peaks at 2θ = 36.4°, 40.25°, 43.94°, 57.9°, and 75.7°, which belong to the Cu and Cu_2_O present, indicating that a fraction of the Cu_2_SO_4_ was reduced and oxidized during the mixing process. As for the crystalline zone of the PBAT, the increase in the concentration of the CuSO_4_ reinforcements decreased the intensity of the diffraction peaks in PBAT, as occurred for the NCs-PBAT/Cu and NCs-PBAT/Cu|Cu_2_O. Thus, the incorporation of CuSO_4_ into the polymer matrix decreased its crystallization capacity, probably because CuSO_4_ hinders the growth of crystallites. Because no additional information on the XRD spectra of CuSO_4_ in composite materials has been reported, we will have to investigate its behavior in biodegradable polymers. The degree of crystallinity of the matrix was calculated as:

1 $$ {X}_{\mathrm{c}}=\frac{I_{\mathrm{c}}}{I_{\mathrm{c}}+{I}_{\mathrm{a}}} $$

where *I*_c_ is the area of the peaks of the crystalline phase and *I*_c_ + *I*_a_ is the total area under the diffractogram. The degree of crystallinity values for each material is given in Table 1. These results show that the percentage of crystallinity increases as the concentration of Cu-NPs and Cu|Cu_2_O-NPs increases in the PBAT matrix, which is evident with the increase in the intensity of the peaks in the respective diffractograms.

**Table 1:** Percentage of crystallinity of each of the mixtures of PBAT, NCs-PBAT/Cu, NCs-PBAT/Cu|Cu_2_O, and MCs-PBAT/CuSO_4_

Sample	Percentage of crystallinity (%)
PBAT	6.78
NCs-PBAT/Cu 1%	7.24
NCs-PBAT/Cu 3%	8.36
NCs-PBAT/Cu 5%	9.50
NCs-PBAT/Cu|Cu_2_O 1%	7.15
NCs-PBAT/Cu|Cu_2_O 3%	7.84
NCs-PBAT/Cu|Cu_2_O 5%	8.82
NCs-PBAT/CuSO_4_ 1%	6.62
NCs-PBAT/CuSO_4_ 3%	6.28
NCs-PBAT/CuSO_4_ 5%	6.73

On the other hand, the diffractograms show that the nanoparticles did not affect or modify the structure of the PBAT and that the intensity of the peaks is directly proportional to the load percentage of the Cu-NPs and Cu|Cu_2_O-NPs. Moreover, the addition of the CuSO_4_ precursor salt decreased the crystallinity of the polymer compared to that of the polymer in its pure state. This condition occurred because the addition of loads concentration in the nanocomposites increased the relative percentage of crystallinity but decreased the crystallinity of the PBAT, a result that, in general, was reported as a slight increase in the total percentage of crystallinity. The MCs-PBAT/CuSO_4_ loads did not present crystalline peaks in their XRD spectra. Therefore, they did not contribute to the increase in crystallinity but caused a decrease in crystallinity in the polymer chain, which explains the decrease in the total percentage of crystallinity in the composite material. Some studies have shown that metal nanoparticles act as centers of nucleation in the orientation of the polymer chains, which in turn increases the crystallinity of the polymer [43].

The FTIR (Additional file 1: Figure S6) spectra show that the characteristic peaks at different load concentrations are at the same frequency but have different intensities. The spectra show that as the concentration of nanoparticles in the polymer matrix increased, the intensity of the peaks corresponding to NCs-PBAT/Cu and NCs-PBAT/Cu|Cu_2_O increased with respect to the PBAT. Therefore, there was no effective interaction between the chains of the PBAT and the nanoparticles. Had there been interaction, some of the signals in the FTIR spectrum would have been displaced as a result of the interaction of the functional groups of the polymer with the surface of the PBAT [40].

### Mechanical Properties (Tensile Test)

To give multifunctionality to biopolymers, nanomaterials that provide special properties to a nanocomposite are usually incorporated. Their inclusion will change the mechanical properties of the material and the intensity of the changes is directly related to the union of the nanostructure with the polymer network [44]. We conducted tensile tests on the nanocomposites and the composite material. The tensile strength and maximum deformation values are summarized in Table 2.

**Table 2:** Tensile strength of PBAT, NCs-PBAT/Cu, NCs-PBAT/Cu|Cu_2_O, and MCs-PBAT/CuSO_4_

Sample	At break	
	Stress (Mpa)	Strain (%)
PBAT	20.43 ± 4.34	445.50 ± 53.05
NCs-PBAT/Cu 1%	18.80 ± 5.86	414.79 ± 26.63
NCs-PBAT/Cu 3%	21.67 ± 5.25	396.96 ± 18.01
NCs-PBAT/Cu 5%	21.57 ± 2.43	361.69 ± 39.08
NCs-PBAT/Cu|Cu_2_O 1%	21.13 ± 5.22	423.25 ± 31.51
NCs-PBAT/Cu|Cu_2_O 3%	20.09 ± 3.48	432.07 ± 33.71
NCs-PBAT/Cu|Cu_2_O 5%	19.38 ± 4.62	406.41 ± 46.05
NCs-PBAT/CuSO_4_ 1%	19.70 ± 3.73	389.57 ± 61.14
NCs-PBAT/CuSO_4_ 3%	20.69 ± 4.70	434.70 ± 29.27
NCs-PBAT/CuSO_4_ 5%	19.38 ± 2.53	375.37 ± 22.33

**Fig. 3:**
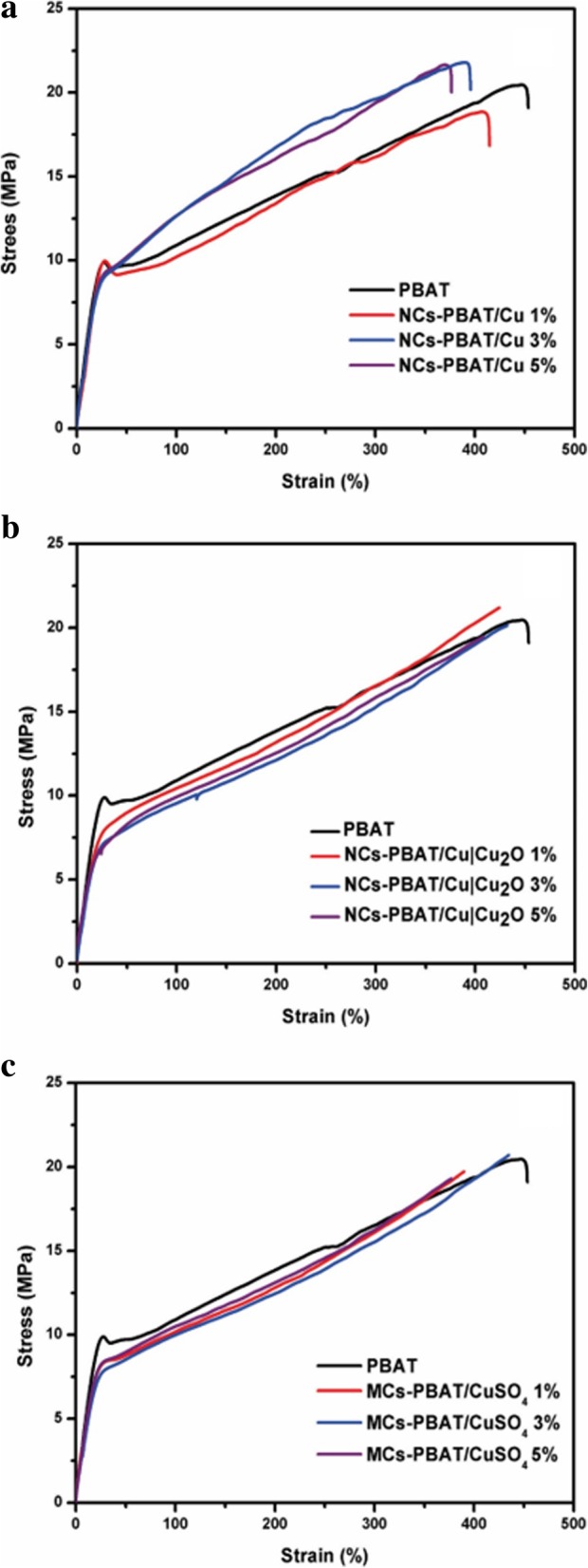
Stress and strain of PBAT, NCs-PBAT/Cu, NCs-PBAT/Cu|Cu_2_O, and MCs-PBAT/CuSO_4_

Figure 3 shows the average curves of the tensile tests on the nanocomposites and composite material. As the permanent deformation of the material began, the effect of the concentration of the nanoparticles in the polymer could be distinguished. Figure 3a shows the results for NCs-PBAT/Cu. The results show that the inclusion of nanostructures did not considerably affect the elastic range but there were noticeable changes in the yield strength. As the concentration of the Cu-NPs increased, maximum resistance increased and maximum elongation decreased. These changes clearly indicate that the nanostructures harden the PBAT. At 3% concentration of Cu-NPs, the tensile strength slightly increased but the elongation percentage in the fracture decreased between 30 and 35%.

Figure 3b shows the results of the tensile tests on the NCs-PBAT/Cu|Cu_2_O. The 1% load nanocomposite clearly showed an increase in tensile strength and elongation with respect to the PBAT. There was no appreciable effect on the elastic range, but it did appear to be above the yield stress. In addition, the curve for the 3% load NCs-PBAT/Cu|Cu_2_O shows there was no significant difference with respect to the PBAT. The same behavior is seen with curve for the 5% load NCs-PBAT/Cu|Cu_2_O. The curves for MCs-PBAT/CuSO_4_ (Fig. 3c) show that the yield stress decreased for the three concentrations of CuSO_4_ with respect to the PBAT.

From the results, we can conclude that the reinforcements did not significantly change the mechanical properties of the PBAT. Venkatesan and Rajeswari [45] showed a significant increase in mechanical properties by incorporating ZnO nanoparticles in a PBAT matrix with respect to that of the PBAT. Similar results with some improvements were obtained by Chen and Yang [46]. They elaborated a PBAT nanocomposite with montmorillonite nanoparticles using melt blending.

Our investigation found that the NCs-PBAT/Cu|Cu2O 3 and 5% and MCs-PBAT/CuSO4 1 and 5% had slightly decreased tensile strength, that is, there were no significant variations in the mechanical properties. However, the NCs-PBAT/Cu|Cu_2_O 1% and MCs-PBAT/CuSO_4_ 3% had slightly increased tensile strength. Therefore, no reinforcement at any concentration in the matrix caused remarkable variations in the mechanical properties of the PBAT. In addition, as the concentration of Cu-NPs increased, their mechanical properties increased the resistance of the PBAT but elongation could not be maintained. The results of the tensile tests showed that the commercial Cu nanoparticles improved the tensile strength of the PBAT slightly more than did the Cu|Cu_2_O nanoparticles and the CuSO_4_ particles. The difference between the tensile properties found in our investigation and those in the literature could be attributed to load dispersion because the agglomerated particles act as stress concentrators [47]. Finally, the variations in the test values were explained by the preparation conditions of the test samples, the degree of crystallinity of the PBAT, the molecular mass, the degree of interaction at the polymer-reinforcement interface, and the load dispersion because the agglomerates in the matrix could act as stress concentrators.

### Thermal Properties

**Fig. 4:**
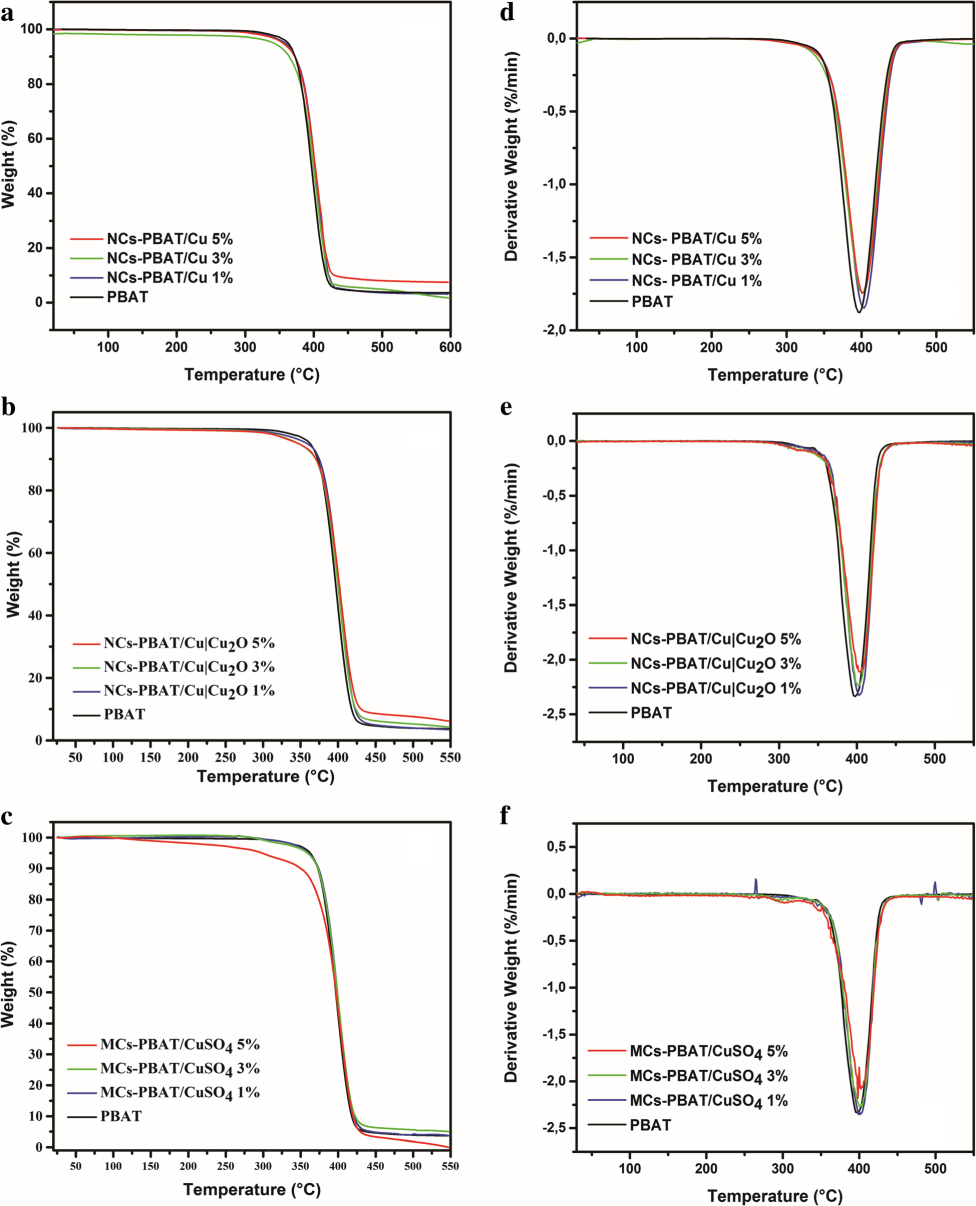
TGA of **a** PBAT and NCs-PBAT/Cu, **b** NCs-PBAT/Cu|Cu_2_O, and **c** MCs-PBAT/CuSO_4_, DTG of **d** PBAT and NCs-PBAT/Cu, **e** NCs-PBAT/Cu|Cu_2_O, **f** MCs-PBAT/CuSO_4_

One of the disadvantages of the PBAT is its low thermal stability because the fusion process can degrade its polymer chains [48]. Therefore, the effect of nanometric and micrometric loads on the decomposition of this biopolymer must be investigated. TGA of NCs-PBAT/Cu, NCs-PBAT/Cu|Cu_2_O, and MCs-PBAT/CuSO_4_ was carried out to observe the changes in the thermal stability of the PBAT caused by the presence of Cu nanoparticles in the matrix. The TGA results are shown in Fig. 4, and the initial (*T*_di_) and final (*T*_df_) decomposition temperatures of the analyzed samples are summarized in Table 3. The thermograms show that the polymer without any load had a weight loss of 1% at 420.77 °C, while the nanocomposites NCs-PBAT/Cu 1, 3, and 5% presented a weight loss of around 3% (Fig. 4a). This suggests that the presence of Cu-NPs at concentrations of 3 and 5% slightly increases the thermal stability of the nanocomposites compared to that of the unloaded polymer. After the final thermal decomposition, the degradation percentages, at around 420–427 °C, of the PBAT matrix and nanocomposites NCs-PBAT/Cu 1, 3, and 5% were 98.9, 97.5, 95.4, and 96.8%, respectively. The residues were higher for Cu-NPs-incorporated nanocomposite samples. Similar results have been reported for PBAT nanocomposites with different loads of Ag-NPs [49].

**Table 3:** Degradation temperature of PBAT, NCs-PBAT/Cu, NCs-PBAT/Cu|Cu_2_O, and MCs-PBAT/CuSO_4_

Sample	Initial degradation temperature *T*_di_ [°C]	Final degradation temperature *T*_df_ [°C]	% of degradation
PBAT	316.21	420.77	98.92
NCs-PBAT/Cu 1%	337.63	425.83	97.58
NCs-PBAT/Cu 3%	341.38	427.17	95.48
NCs-PBAT/Cu 5%	342.72	427.17	96.80
NCs-PBAT/Cu|Cu_2_O 1%	352.36	403.6	92.94
NCs-PBAT/Cu|Cu_2_O 3%	353.24	402.27	92.66
NCs-PBAT/Cu|Cu_2_O 5%	338.12	403.15	90.25
MCs-PBAT/CuSO_4_ 1%	360.91	401.65	94.12
MCs-PBAT/CuSO_4_ 3%	352.73	401.33	92.12
MCs-PBAT/CuSO_4_ 5%	351.52	396.14	94.71

Although no significant change is seen among the curves in Fig. 4b for the NCs-PBAT/Cu|Cu_2_O, the results show that as the Cu|Cu_2_O-NPs increased in the polymer structure, *T*_di_ increased and *T*_df_ decreased with respect to the initial and final degradation temperatures of PBAT; in addition, the total mass loss decreased. By calculating the derivative of the mass with respect to the temperature, we obtained the curves in Fig. 4d–f for the indicated peaks of the nanocomposite with Cu|Cu_2_O-NPs and found that *T*_df_, at which the maximum decomposition occurs, was between 402 and 403 °C (Table 3).

The CuSO_4_ loads incorporated into the polymer matrix, i.e., MCs-PBAT/CuSO_4_, yielded the same behavior as that of the NCs-PBAT/Cu|Cu_2_O, with an increase in *T*_di_ and a decrease in *T*_df_ with respect to the PBAT polymer. The *T*_di_ values of the NCs-PBAT/Cu|Cu_2_O and the MCs-PBAT/CuSO_4_ were greater than that of the NCs-PBAT/Cu, but the *T*_df_ and degradation percentage values were less than those of the nanocomposites with Cu-NPs loads.

This enhancement of the thermal stability of the PBAT is attributed to the barrier effect of the loads. The loads were also supposed to have a shielding effect on the matrix to slow the rate of mass loss of the decomposition product [50]. The data obtained by our analysis were compared with published results to verify that the indicated behavior is usual for this type of polymer. Sinha Ray et al. [51] found by thermal analysis of PBAT reinforced with nanoclays that the degradation temperatures of the nanocomposites were greater than or at least equal to that of the PBAT. In general, the reinforcements improve the thermal stability of the polymer matrix because they act as a heat barrier, which improves the total thermal stability of the system. However, the studies of Sinha Ray et al. and this investigation showed that the thermal stability of the nanocomposite and PBAT compounds only slightly improved. To explain the relatively low improvement in the thermal stability of some nanocomposites, Sinha Ray et al. assumed that in the early stages of thermal decomposition, the reinforcements displace the decomposition to higher temperatures, but in a second stage, the clay layers accumulate heat and then act as a source of heat. This heat source, along with the heat flow supplied by the external heat source, promotes the acceleration of decomposition. This could explain the behavior of the reinforcements in the NCs-PBAT/Cu|Cu_2_O and MCs-PBAT/CuSO_4_. Thus, we conclude that the thermal properties of the nanocomposites and the composite material slightly improve but not significantly. On the other hand, the results of DSC (Additional file 1: Figure S7 and Table S1) indicated that the addition of reinforcements to the matrix slightly hindered the kinetics and degree of crystallization of the PBAT. The addition of clays increased the crystallization temperature from 1 to 10 °C and the melting temperature from 1 to 5 °C. These phenomena were probably due to an increase in the viscosity of the polymer with the addition of clays, which reduced the mobility of the macromolecular chains against the growth of crystals.

### Comparative Evaluation of the Antimicrobial Activity of NCs-PBAT/Cu, NCs-PBAT/Cu|Cu_2_O, and MCs-PBAT/CuSO_4_

#### Qualitative Test

After the experimental procedure was performed, we wanted to observe whether bacterial colonies were inhibited by each PBAT sample, i.e., NCs-PBAT/Cu 1, 3, and 5%; NCs-PBAT/Cu|Cu_2_O 1, 3, and 5%; and MCs-PBAT/CuSO_4_ 1, 3, and 5%. We decided to use the 3% concentrations because the 1% concentrations did not produce enough bacterial inhibition and the 5% concentration produced behavior similar to that of the 3% concentration, the minimum percentage with activity that avoided toxicity in the polymer.

#### Quantitative Test

**Fig. 5:**
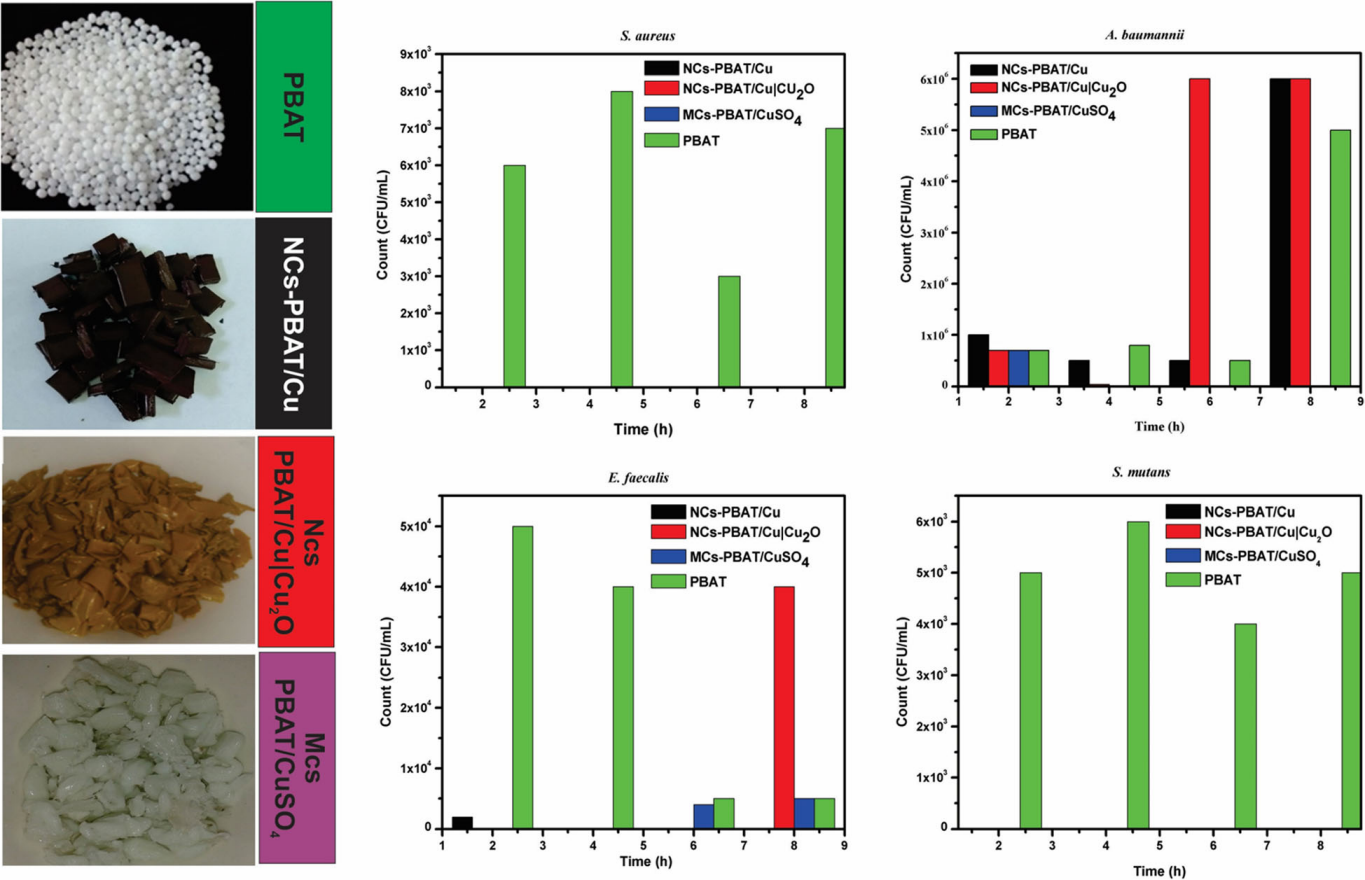
Bacterial activity and colonization count PBAT, NCs-PBAT/Cu-3%, NCs-PBAT/Cu|Cu_2_O 3%, and MCs-PBAT/CuSO_4_ 3% for each strain of bacteria. *Staphylococcus aureus*, *Acinetobacter baumanni*, *Enterococcus faecalis*, *Streptococcus mutans*

**Fig. 6:**
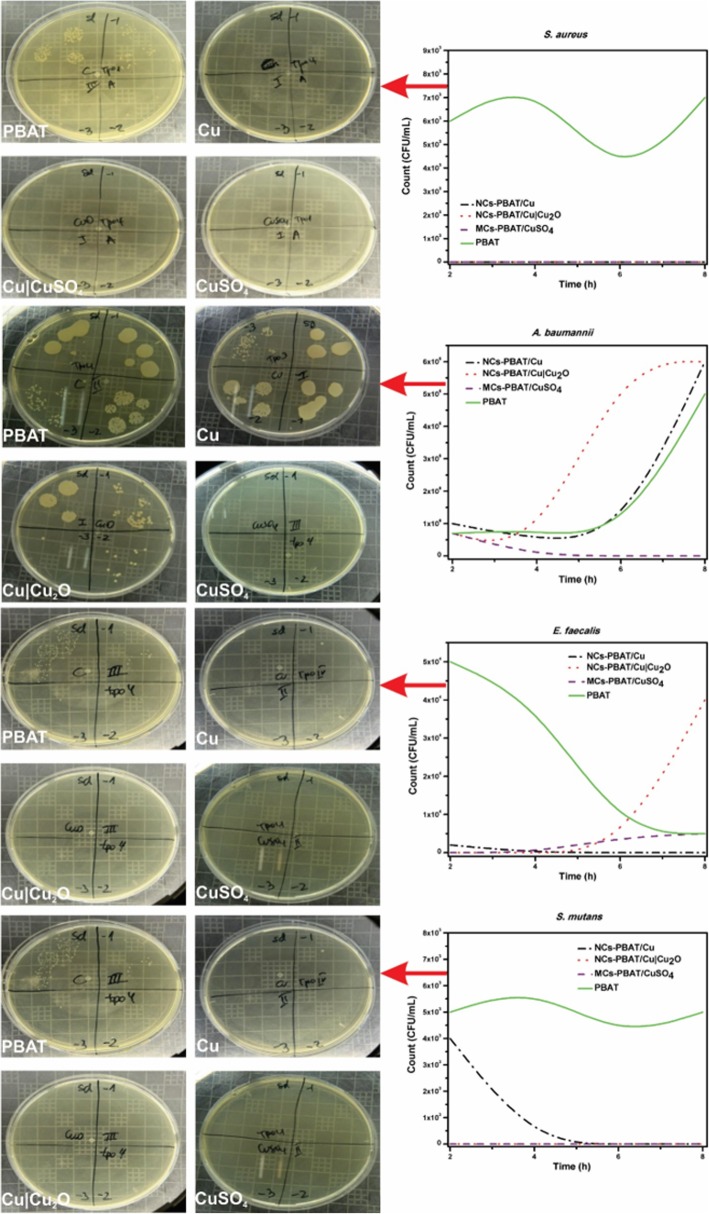
Graphical analysis of colony count (CFU/mL) vs time (h) of PBAT, NCs-PBAT/Cu-3% NCs-PBAT/Cu|Cu_2_O 3%, and MCs-PBAT/CuSO_4_ 3% for each strain of bacteria. *Enterococcus faecalis*, *Acinetobacter baumanni*, *Streptococcus mutans*, *Staphylococcus au*reus

The study was carried out at different contact times using four bacterial strains and the PBAT samples NCs-PBAT/Cu 3%, NCs-PBAT/Cu|Cu_2_O 3%, and MCs-PBAT/CuSO_4_ 3%. The times and colony-forming unit counts (CFU/mL) are presented in Table 4, and the bacterial activity and colony count for each Petri dish are shown in Fig. 5. In addition, a graphical analysis is shown in Fig. 6, where images of bacterial growth are also presented. The statistical analysis of the data is summarized in Table 5.

**Table 4:** Bacterial colonies count corresponding to four incubation times for each sample of NCs-PBAT/Cu-3%, NCs-PBAT/Cu|Cu_2_O 3%, and MCs-PBAT/CuSO_4_ 3% and PBAT

Time (h)	*Acinetobacter baumanni*				*Enterococcus faecalis*				*Streptococcus mutans*				*Staphylococcus aureus*			
	PBAT	NCs-PBAT/Cu 3%	NCs-PBAT/Cu|Cu_2_O 3%	MCs-PBAT/CuSO_4_ 3%	PBAT	NCs-PBAT/Cu 3%	NCs-PBAT/Cu|Cu_2_O 3%	MCs-PBAT/CuSO_4_ 3%	PBAT	NCs-PBAT/Cu 3%	NCs-PBAT/Cu|Cu_2_O 3%	MCs-PBAT/CuSO_4_ 3%	PBAT	NCs-PBAT/Cu 3%	NCs-PBAT/Cu|Cu_2_O 3%	MCs-PBAT/CuSO_4_ 3%
Time I	7 × 10^5^	1 × 10^6^	7 × 10^5^	7 × 10^5^	5 × 10^4^	2 × 10^3^	0	0	5 × 10^3^	4x10^3^	0	0	6 × 10^3^	0	0	0
Time II	8 × 10^5^	5 × 10^5^	4 × 10^4^	3 × 10^3^	4 × 10^4^	0	0	0	6 × 10^3^	0	0	0	8 × 10^3^	0	0	0
Time III	5 × 10^5^	5 × 10^5^	6 × 10^6^	1 × 10^2^	5 × 10^3^	0	0	4 × 10^3^	4 × 10^3^	0	0	0	3 × 10^3^	0	0	0
Time IV	5 × 10^6^	6 × 10^6^	6 × 10^6^	0	5 × 10^3^	0	4 × 10^4^	5 × 10^3^	5 × 10^3^	0	0	0	7 × 10^3^	0	0	0

**Table 5:** Statistical analysis for each bacterial strain

Sample	*Acinetobacter baumanni*		*Enterococcus faecalis*		*Streptococcus mutans*		*Staphylococcus aureus*	
	Average	Standard deviation	Average	Standard deviation	Average	Standard deviation	Average	Standard deviation
PBAT	1.75 × 10^6^	2.17 × 10^6^	2.5 × 10^4^	2.35 × 10^4^	5.0 × 10^3^	7.1 × 10^2^	6.0 × 10^3^	1.8 × 10^3^
NCs-PBAT/Cu 3%	2.0 × 10^6^	2.68 × 10^6^	5.0 × 10^2^	1.0 × 10^3^	8.0 × 10^2^	1.8 × 10^3^	0	0
NCs-PBAT/Cu|Cu_2_O 3%	3.19 × 10^6^	3.26 × 10^6^	1.0 × 10^4^	2.0 × 10^3^	0	0	0	0
MCs-PBAT/CuSO_4_ 3%	1.76 × 10^5^	3.49 × 10^5^	2.25 × 10^3^	2.63 × 10^3^	0	0	0	0

The study of *A. baumannii* found that the colonies grew in all periods (2, 4, 6, and 8 h) in the samples containing Cu-NPs, Cu|Cu_2_O-NPs, and PBAT. High bactericidal activity occurred with the sample containing CuSO_4_ during exposure times of 4, 6, and 8 h, decreasing from 7 × 10^5^ to 0 CFU/mL. The sample containing Cu-NPs showed a significant increase in the growth of bacterial colonies from 1 × 10^5^ to 6 × 10^6^ CFU/mL, with an average of 2 × 10^6^ CFU/mL. The bacterial colonies in the sample containing Cu|Cu_2_O-NPs grew from 7 × 10^5^ in time I to 6 × 10^6^ in time IV, with an average growth of 3.19 × 10^6^ CFU/mL. Bacterial growth in the PBAT reached an average of 1.75 × 10^6^ CFU/mL.

The study of *E. faecalis* found good bactericidal activity by the samples containing Cu-NPs, Cu|Cu_2_O-NPs, and CuSO_4_, with average colony growth of 5 × 10^2^, 1 × 10^4^, and 2.2 × 10^3^ CFU/mL, respectively, while the PBAT did not show bactericidal activity and the colonies grew at all times. Colony growth in the sample containing Cu-NPs was 2 × 10^3^ CFU/mL at 2 h then dropped to zero at 4, 6, and 8 h, whereas the samples containing Cu|Cu_2_O-NPs had 0 CFU/mL at times I, II, and III, but 4 × 10^4^ CFU/mL at time IV. Samples containing CuSO_4_ prevented the growth of bacteria in times I and II with growth activity of 0 CFU/mL, but colonies grew to 4 × 10^3^ and 5 × 10^3^ CFU/mL for times III and IV, respectively. PBAT did not show bactericidal activity against *E. faecalis*.

The study of *S. mutans* found no colony growth in the samples containing Cu|Cu_2_O-NPs and CuSO_4_. The sample containing Cu-NPs showed very good bactericidal activity except at time I, at which colony growth was 4 × 10^3^ CFU/mL, making the average growth for the four times 8 × 10^2^ CFU/mL. PBAT without reinforcement showed no bactericidal activity against *S. mutans*. The samples containing Cu-NPs, Cu|Cu_2_O-NPs, and CuSO_4_ in contact with *S. aureus* showed an excellent bactericidal response. They completely inhibited the growth of colonies, while PBAT did not show any bactericidal activity against *S. aureus*, which grew an average of 6 × 10^3^ CFU/mL.

In general, the antibacterial effectiveness of polymer-and-metal nanocomposites improves with a high surface/volume ratio, which increases the number of ions released from the nanoparticles into the polymer. The mechanism of the corrosion of Cu in aqueous solutions and the resulting Cu species vary with pH. In general, the species Cu_2_O and CuO are formed and can be dissolved in Cu ions. Elemental metal particles require the presence of water and oxygen molecules to release a small amount of ions. Therefore, retention of water and oxygen within the polymer is crucial for the release of Cu ions. Some properties of polymer-and-metal nanocomposites such as the crystallinity and polarity of the matrix, which constitute a barrier for the diffusion of water molecules and ions during their propagation, can affect the rate of release. Shankar and Rhim [49] prepared films composed of PBAT and Ag nanoparticles (PBAT/Ag-NPs) that showed strong antibacterial activity against *E. coli* and *Listeria monocytogenes* compared with that of PBAT films without Ag-NPs. Similar results were obtained by Venkatesan and Rajeswari [45] when they evaluated the antimicrobial activity of ZnO-NPs incorporated in a PBAT matrix. The PBAT compound, which was used as a control matrix, showed no antimicrobial activity compared to the PBAT/ZnO-NPs nanocomposite films. The results showed that the films had high bactericidal activity against the pathogens tested (*E. coli* and *S. aureus*), with increased inhibition of bacterial growth as the ZnO load concentration increased from 1 to 10% by weight. This ability of Cu, Zn, and Ag nanoparticles to inhibit bacterial growth is mainly due to the irreparable damage to the membrane of the bacterial cells caused by the interaction between the surface of the bacteria and these oxides and metals [52, 53]. Compared with the works discussed above, our investigation found significant antimicrobial activity against inpatient and oral-resistant strains.

To complement this investigation, we performed water absorption tests using three different media and following point 7.4, “Long-Term Immersion”, in ASTM D570-98. The results of these tests are reported in the supplementary material, Additional file 1: Table S2–S4 and Figure S8, with their respective analysis. Analysis showed that sulfate-based composite materials absorb large amounts of water, even in acidic and basic environments. This phenomenon greatly affects the mechanical properties of these materials; however, resistant bacteria, such as *A. baumannii*, require an immediate Cu^+^ distribution to control them. This explains the antimicrobial power of CuSO_4_ within the PBAT matrix.

## Conclusions

Using XRD and TEM, we determined that the synthesis of nanocomposites and material composites based on PBAT using chemical reduction and a mixture of metal Cu nanoparticles with CuO_2_, where Cu had a spherical morphology and Cu_2_O had a polyhedral morphology. The structural characterization of the NCs and MCs by FTIR and XRD showed that the Cu-NPs, Cu|Cu_2_O-NPs, and CuSO_4_ reinforcements did not modify the structure of the PBAT. However, they did slightly alter the percentage of its crystallinity, which increased with NPs and decreased with CuSO_4_. On the other hand, the mechanical properties of the PBAT for both the NCs and MCs did not vary significantly with the addition of reinforcements, meaning that the PBAT maintained its mechanical properties. From the thermal tests, we concluded that reinforcing the PBAT did not fundamentally improve its thermal properties, it only increased its thermal stability a few degrees Celsius, which is not significant. Antimicrobial analyses showed that the Cu|Cu_2_O-NPs within the PBAT generated antibacterial activity against *E. faecalis* and *S. mutans* and excellent bactericidal properties against *S. aureus*. CuSO_4_ had a good bactericidal response against *A. baumannii*, *E. faecalis*, and *S mutans* and an exceptional response against *S. aureus*. The PBAT without loads did not present bactericidal properties when in contact with the bacterial strains. In general, the addition of loads into the PBAT generates bactericidal activity that the polymer does not possess by itself. The addition of CuSO_4_ yielded the best antimicrobial response against the four strains used in this investigation. In the search for new applications for bionanocomposites, it will be essential to evaluate their antimicrobial response in food containers, medical devices, packaging, and other products; analyze their biocidal effects against other bacteria against which only NPs have antibacterial characteristics; and justify the expense associated with their synthesis.

## Additional file

Additional file 1: Supplementary figures and tables. This file contains supplementary **Figures S1–S8.** and **Tables S1–S4.**. (DOCX 1716 kb)

## Abbreviations

Cu|Cu_2_O-NPs: Copper/cuprous oxide nanoparticles
Cu-NPs: Copper nanoparticles
CuSO_4_: Copper sulfate
DSC: Differential scanning calorimetry
FTIR: Fourier transform infrared spectroscopy
MC: Composite material
MCs-PBAT/CuSO_4_: Composite materials of poly(butylene adipate-co-terephthalate) with copper sulfate
NCs: Nanocomposites
NCs-PBAT/Cu: Nanocomposites of poly(butylene adipate-co-terephthalate) with copper nanoparticles
NCs-PBAT/Cu|Cu_2_O: Nanocomposites of poly(butylene adipate-co-terephthalate) with copper/cuprous oxide nanoparticles
PBAT: Poly(butylene adipate-co-terephthalate)
TEM: Transmission electron microscopy
TGA: Thermogravimetric analysis
XRD: X-ray diffraction
