# Supplementary material for: Comparative Study of the Antimicrobial Effect of Nanocomposites and Composite Based on Poly(butylene adipate-co-terephthalate) Using Cu and Cu/Cu2O Nanoparticles and CuSO4
Source: Nanoscale Res Lett. 2019 May 9;14:158. doi: 10.1186/s11671-019-2987-x (PMC6509317; doi:10.1186/s11671-019-2987-x)
Supplement: Supplementary file 1 — Supplementary figures and tables. This file contains supplementary Figures S1–S8. and Tables S1–S4.. (DOCX 1716 kb) [file 11671_2019_2987_MOESM1_ESM.docx]

**Additional file 1**

**Comparative study of the antimicrobial effect of nanocomposites and composite based on poly(butylene adipate-co-terephthalate) using Cu and Cu/Cu_2_O nanoparticles and CuSO_4_**

**A. F. Jaramillo^1^, S. A. Riquelme^2^, G. Sánchez-Sanhueza^3^,**

**C. Medina^4^, E. Pérez-Tijerina^5^, F. Solís-Pomar^5^, D. Rojas^6^, C. Montalba^7^ and M. F. Melendrez^6*^**


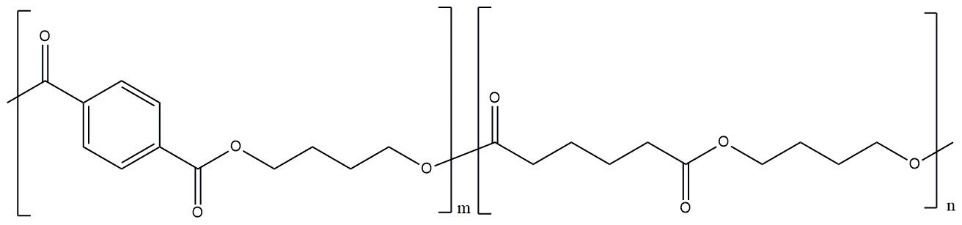


Figure S1: Molecular structure poly (butylene adipate-co-terephthalate) (PBAT).


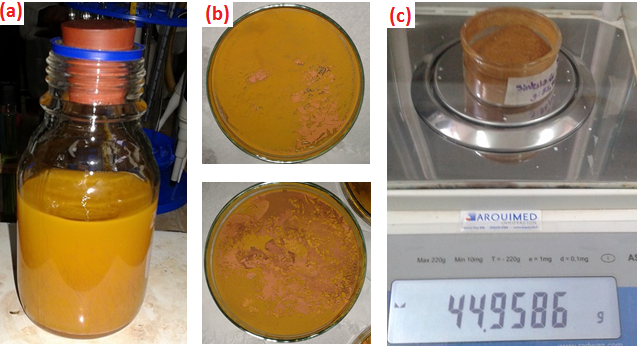


Figure S2: (a) Final solution ocher tonality. (b) Cu|Cu_2_O-NPs dried in Petri dishes, (c) Cu|Cu_2_O-NPs powder.


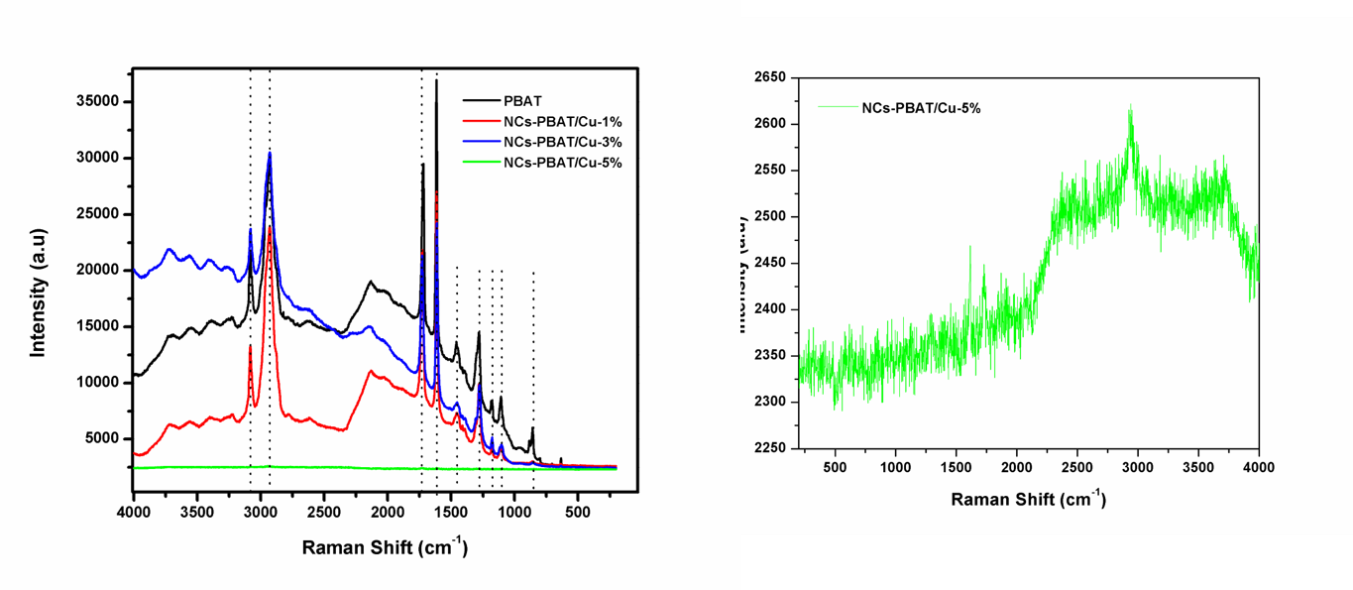


Figure S3. Spectra of Raman spectroscopy of the nanocomposites based on PBAT and Cu-NPs.


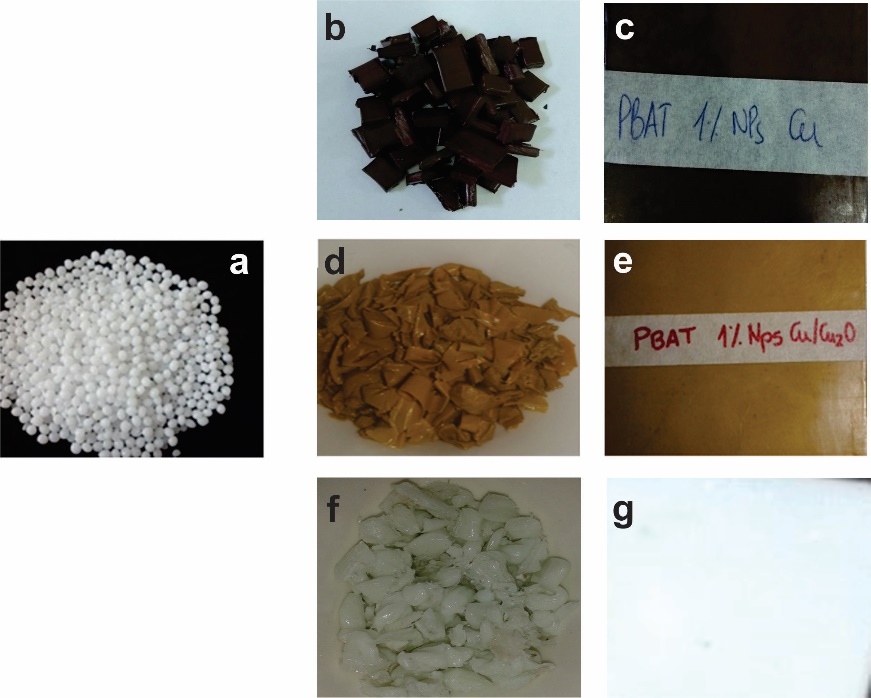


Figure S4: a) PBTA, b) NCs-PBAT/Cu crushed, c) Pressed test piece of NCs-PBAT/Cu, d) NCs-PBAT/Cu|Cu_2_O crushed, e) Pressed test piece of PBAT/Cu|Cu_2_O, f) MCs-PBAT/CuSO_4_ crushed, g) Pressed test piece of MCs-PBAT/CuSO_4_.


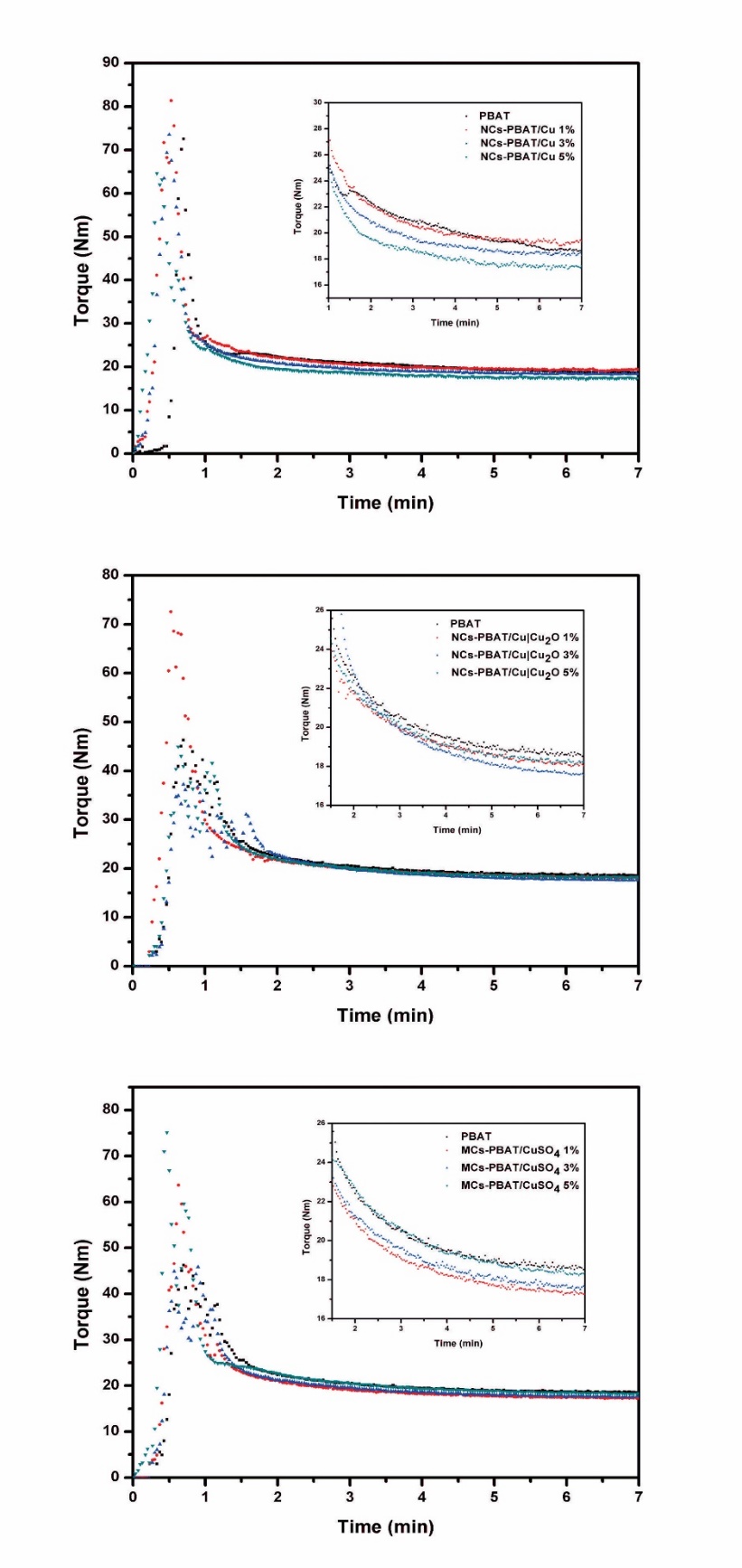


Figure S5: Torque variation of each NCs-PBAT/Cu, NCs-PBAT/Cu|Cu_2_O and MCs-PBAT/CuSO_4_.


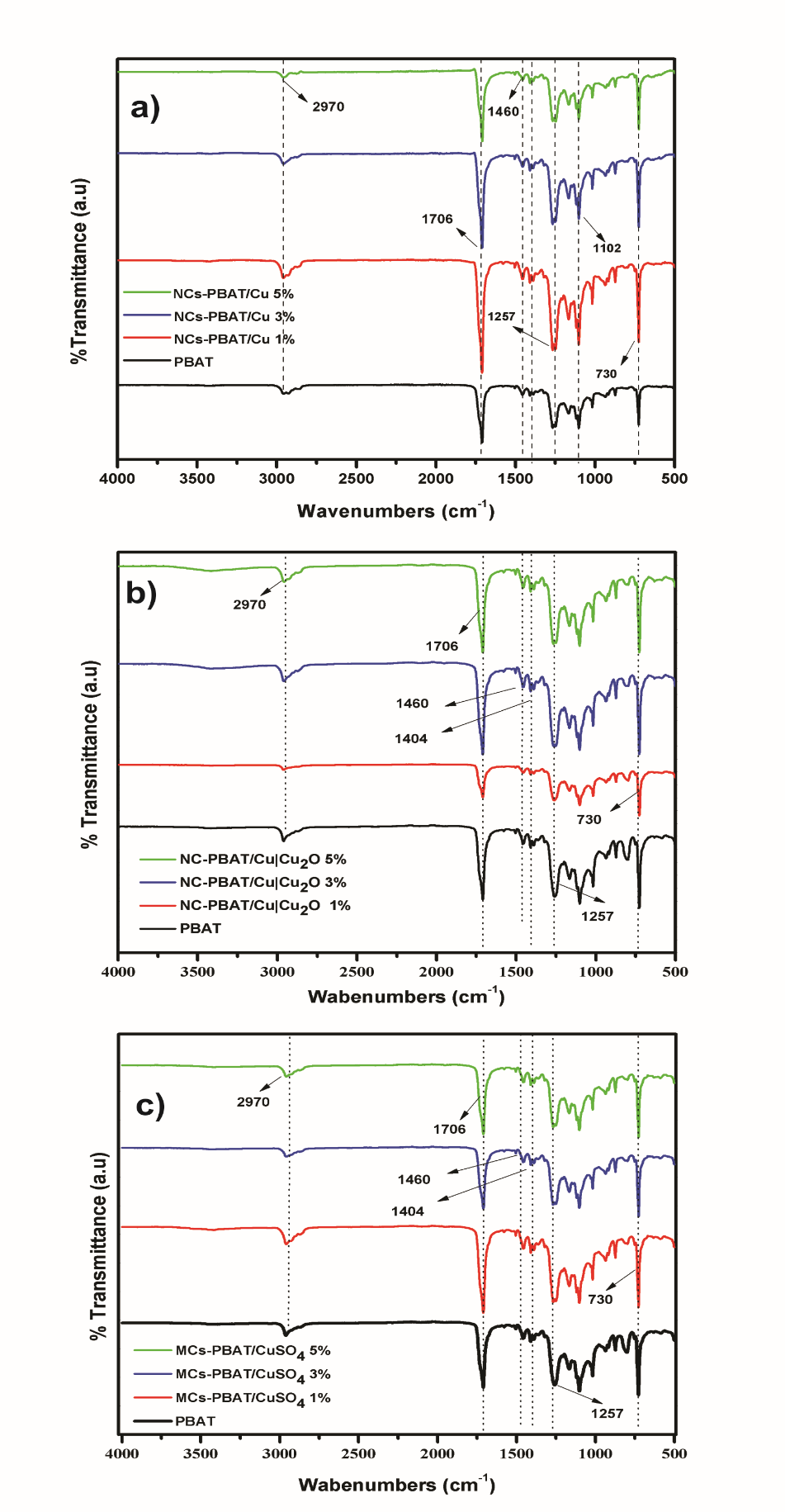


Figure S6: FTIR spectra of PBAT, NCs-PBAT/Cu, NCs-PBAT/Cu|Cu_2_O and MCs-PBAT/CuSO_4_

The vibrations of the bonds of the PBAT and their variation with the addition of nanometric loads (Cu-NPs and Cu|Cu_2_O-NPs) and micrometric loads (CuSO_4_) were verified by FTIR analysis. Fig. S6 shows the infrared spectrum of the PBAT and the peaks representative of the functional group’s characteristic of the polymer around 2970, 1706, 1460‑1404, 1257, and 730 cm^−1^. The peak at 2970 cm^−1^ corresponds to the stretching of the CH and CH_2_ bonds in aliphatic and aromatic compounds. The peak at 1706 cm^−1^ corresponds to the vibrations of the C=O ester carbonyl group. In the region of 1460‑1400 cm^−1^, there are characteristic flexion bands of the C=C bonds of the aromatic ring. At ~1257 cm^‑1^, there is an asymmetric vibration of C−O bonds of the ester, and at 730 cm^−1^, there is an acute peak due to adjacent methylene groups (CH single bond) [1]. In a similar manner, the FTIR spectra for the three concentrations of the MCs-PBAT/CuSO_4_ were analyzed (Fig. S6c) and the same peaks corresponding to the PBAT were found. However, a slight variation in the intensity of the peaks is clearly observed in Fig. S6c. This shows that the Cu-NPs, Cu|Cu_2_O-NPs, and CuSO_4_ interact with the chains of the PBAT.

**
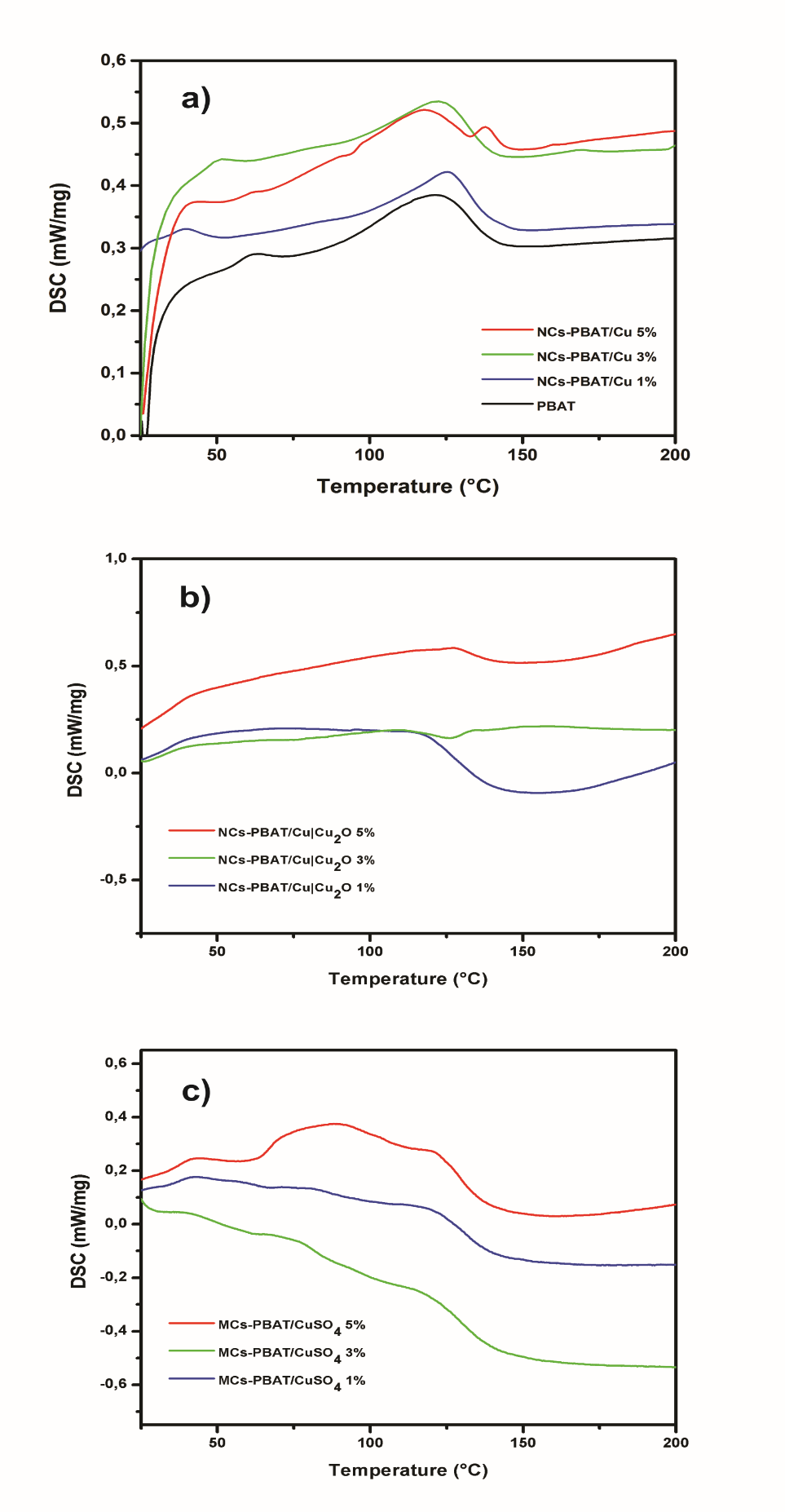
**

Figure S7**:** DSC of: a) PBAT and NCs-PBAT/Cu, b) NCs-PBAT/Cu|Cu_2_O, c) MCs-PBAT/CuSO_4_.

Another way to monitor polymer changes due to nanomaterial loads is by analyzing the thermal transitions, i.e., obtain the thermal behavior in the glass (*T*_g_) and fusion (*T*_m_) transitions of the nanocomposites at their different concentrations and compare it to that of the PBAT matrix. Fig. S7a shows the melting temperatures (*T*_m_) of the different nanocomposites. The PBAT matrix had a *T*_m_ of 120°C, which was 3‑6°C lower with the incorporation of Cu-NPs. The graphs of Fig. S7 show that the peaks corresponding to endothermic transitions associated with the fusion processes appear between 121 and 127 C (see Table S8). Results shows that the increase in Cu-NPs concentration did not produce any significant change in the PBAT chain, mainly because heterogeneous nucleation in the presence of Cu-NPs increases the nucleation sites in the polymer matrix. This phenomenon occurs mainly because the Cu-NPs act as a barrier to heat, thus slightly increasing the thermal stability of the system [2].

A small peak at ~68 C represented the crystallization zone of the PBAT. The peak at 121.42 C represented the melting point, which agreed with the theoretical range of *T*_m_ for the PBAT. The glass transition zone does not appear on the Fig.S7 because the test started at 20 C and *T*_g_ for the PBAT is ‑30°C [3]. Fig. S7b shows the thermal curves for the NCs-PBAT/Cu|Cu_2_O 1, 3, and 5%, where variation in the melting points is seen. The melting points of NCs-PBAT/Cu|Cu_2_O 1 and 3% decreased by approximately 11°C and that of NCs-PBAT/Cu|Cu_2_O 5% slightly increased with respect to PBAT. It was not possible to distinguish the crystallization peaks in the curves for NCs-PBAT/Cu|Cu_2_O 1, 3, and 5%. These data lead us to conclude that the incorporation of nanometric loads into the PBAT matrix did not significantly affect the thermal properties of the polymer because its melting point stayed within the theoretical range

Fig. S7c shows the curves for the MCs-PBAT/CuSO_4_ 1, 3, and 5%. As the concentration of CuSO4 in the PBAT matrix increased, the melting point of the polymer decreased approximately 2 C (Table S8). From this, we deduced that the thermal properties of the polymer did not change significantly with the addition of loads. The curve for the MCs-PBAT/CuSO_4_ 5% showed a decrease in crystallization temperature, that is, the polymer crystallized more rapidly at the higher concentration of CuSO4 in the matrix. Chivrac et al. [4] and Lee et al. [5] reported similar results for a PBAT nanocomposite with clay loads.

Table S1**:** Melting temperature of PBAT, NCs-PBAT/Cu, NCs-PBAT/Cu|Cu_2_O and MCs-PBAT/CuSO_4_.

| **Sample** | **Melting point T_m_ (°C)** |
| --- | --- |
| **PBAT** | 121.42 |
|  | |
| **NCs-PBAT/Cu 1%** | 125.64 |
| **NCs-PBAT/Cu 3%** | 122.64 |
| **NCs-PBAT/Cu 5%** | 117.88 |
|  | |
| **NCs-PBAT/Cu\|Cu_2_O 1%** | 110.77 |
| **NCs-PBAT/Cu\|Cu_2_O 3%** | 110.39 |
| **NCs-PBAT/Cu\|Cu_2_O 5%** | 128.27 |
|  | |
| **MCs-PBAT/CuSO_4_ 1%** | 118.86 |
| **MCs-PBAT/CuSO_4_ 3%** | 122.60 |
| **MCs-PBAT/CuSO_4_ 5%** | 120.54 |

ASTM D570-98 was used as a reference for the water absorption tests. The procedure followed section 7.4, “Long-Term Immersion”. Samples were taken from PBAT, NCs-PBAT/Cu|Cu_2_O 1, 3, and 5%, and MCs-PBAT/CuSO_4_ 1, 3, and 5%. For the procedure, 21 samples (40 × 40 × 1 mm^3^) were cut and dried in an oven at 50 °C for 24 h to remove all moisture from the samples. They were then weighed and their masses were recorded. The samples were placed in Petri dishes containing different media. Seven samples were analyzed in water, seven in NaOH (pH 14), and seven in HCl (pH 1.5). The samples were weighed at 24 h and 7, 14, and 28 days. Absorption was calculated according to the mass recorded and the number of days of exposure to an immersion medium. The difference between the initial weight of the polymer (dry) and the weight of the wet polymer was the amount of water absorbed. The percentage of weight gain for each sample was calculated using equation 2 and the results are presented in Tables S6‑S8.

| $Weight gain \left( \% \right)= \frac{Initial dry weight-wet weight}{Initial dry weight} x 100$ | Ec. 2 |
| --- | --- |

Table S2: Weight increase (%) of the polymer in water

| **Water** | **Initial mass** | **Final mass** | **Weight gain (%)** |
| --- | --- | --- | --- |
| **PBAT** | 2.33 | 2.34 | 0.26 |
| **NCs-PBAT/Cu\|Cu_2_O 1%** | 1.96 | 1.97 | 0.7 |
| **NCs-PBAT/Cu\|Cu_2_O 3%** | 2.33 | 2.35 | 0.91 |
| **NCs-PBAT/Cu\|Cu_2_O 5%** | 2.49 | 2.52 | 1.17 |
| **MCs-PBAT/CuSO_4_ 1%** | 2.07 | 2.11 | 1.81 |
| **MCs-PBAT/CuSO_4_ 3%** | 2.1 | 2.17 | 3.54 |
| **MCs-PBAT/CuSO_4_ 5%** | 2.13 | 2.23 | 4.68 |

Table S3: Weight gain (%) of the polymer in NaOH

| **NaOH** | **Initial mass** | **Final mass** | **Weight gain (%)** |
| --- | --- | --- | --- |
| **PBAT** | 2.34 | 2.36 | 0.67 |
| **NCs-PBAT/Cu\|Cu_2_O 1%** | 2.19 | 2.21 | 0.76 |
| **NCs-PBAT/Cu\|Cu_2_O 3%** | 2.22 | 2.24 | 0.74 |
| **NCs-PBAT/Cu\|Cu_2_O 5%** | 2.48 | 2.5 | 0.97 |
| **MCs-PBAT/CuSO_4_ 1%** | 2.15 | 2.17 | 1.08 |
| **MCs-PBAT/CuSO_4_ 3%** | 1.99 | 2.07 | 3.4 |
| **MCs-PBAT/CuSO_4_ 5%** | 2.16 | 2.27 | 5.16 |

Table S4: Weight gain (%) of the polymer in HCl

| **HCl** | **Initial mass** | **Final mass** | **Weight gain (%)** |
| --- | --- | --- | --- |
| **PBAT** | 2.36 | 2.39 | 1.46 |
| **NCs-PBAT/Cu\|Cu_2_O 1%** | 2.05 | 2.08 | 0.85 |
| **NCs-PBAT/Cu\|Cu_2_O 3%** | 2.44 | 2.45 | 0.82 |
| **NCs-PBAT/Cu\|Cu_2_O 5%** | 2.58 | 2.61 | 0.91 |
| **MCs-PBAT/CuSO_4_ 1%** | 2.03 | 2.05 | 0.86 |
| **MCs-PBAT/CuSO_4_ 3%** | 2.05 | 2.07 | 0.97 |
| **MCs-PBAT/CuSO_4_ 5%** | 2.33 | 2.36 | 1.25 |

The PBAT absorbed more water as the amount of NPs in its structure increased. The PBAT reinforced with CuSO_4_ absorbed more than the PBAT reinforced with NPs. In addition, its absorption increased as the concentration of CuSO_4_ increased. The saturated polymer showed no cracks or visible deterioration. The results for samples of the PBAT immersed in NaOH were similar to those for samples in water, that is, the PBAT began to absorb more NaOH as the concentration of NPs increased. However, the absorption by the PBAT reinforced with CuSO_4_ was greater. The absorption by the PBAT in HCl was different than that in the other two media. The percentage of acid absorbed decreased for the PBAT with incorporated NPs.


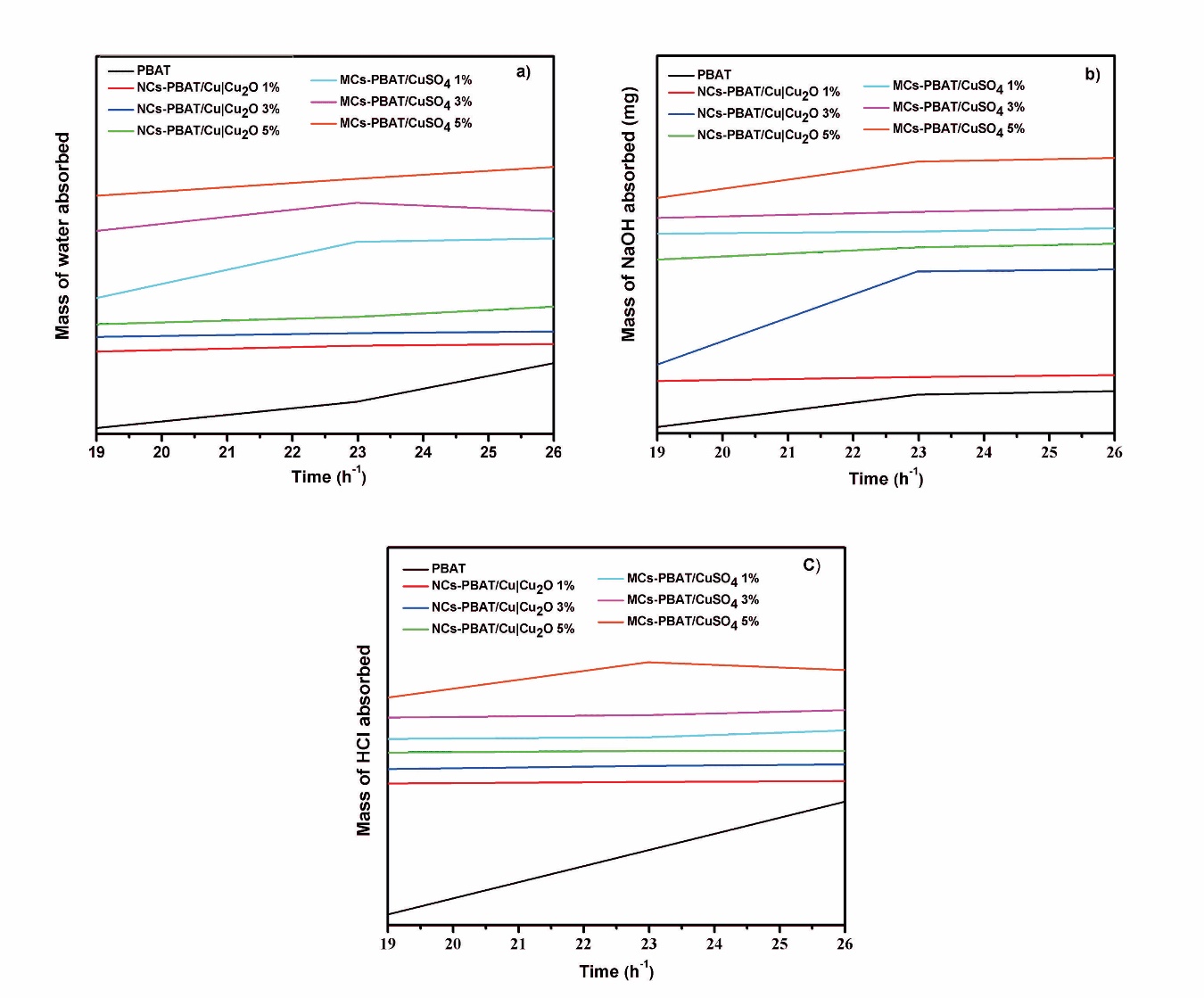


Figure S8: Graph of absorbed mass vs time of NCs and MCs in a) water, b) NaOH, c) HCl

**References of supplementary material**

[1] R. Al-Itry, K. Lamnawar, A. Maazouz, Improvement of thermal stability, rheological and mechanical properties of PLA, PBAT and their blends by reactive extrusion with functionalized epoxy, Polym. Degrad. Stab. 97 (2012) 1898–1914. doi:10.1016/j.polymdegradstab.2012.06.028.

[2] H. Moustafa, H. Galliard, L. Vidal, A. Dufresne, Facile modification of organoclay and its effect on the compatibility and properties of novel biodegradable PBE/PBAT nanocomposites, Eur. Polym. J. 87 (2017) 188–199. doi:10.1016/j.eurpolymj.2016.12.009.

[3] F. Signori, M.B. Coltelli, S. Bronco, Thermal degradation of poly(lactic acid) (PLA) and poly(butylene adipate-co-terephthalate) (PBAT) and their blends upon melt processing, Polym. Degrad. Stab. 94 (2009) 74–82. doi:10.1016/j.polymdegradstab.2008.10.004.

[4] F. Chivrac, E. Pollet, L. Avérous, Nonisothermal crystallization behavior of poly(butylene adipate-co-terephthalate)/clay nano-biocomposites, J. Polym. Sci. Part B Polym. Phys. 45 (2007) 1503–1510. doi:10.1002/polb.21129.

[5] S.-R. Lee, H. Park, H. Lim, T. Kang, X. Li, W.-J. Cho, C.-S. Ha, Microstructure, tensile properties, and biodegradability of aliphatic polyester/clay nanocomposites, Polymer (Guildf). 43 (2002) 2495–2500. doi:10.1016/S0032-3861(02)00012-5.
